# Synthesis, characterization and C–H amination reactivity of nickel iminyl complexes†

**DOI:** 10.1039/c9sc04879k

**Published:** 2019-12-11

**Authors:** Yuyang Dong, James T. Lukens, Ryan M. Clarke, Shao-Liang Zheng, Kyle M. Lancaster, Theodore A. Betley

**Affiliations:** Department of Chemistry and Chemical Biology, Harvard University 12 Oxford Street Cambridge Massachusetts 02138 USA betley@chemistry.harvard.edu; Department of Chemistry and Chemical Biology, Baker Laboratory, Cornell University Ithaca New York 14853 USA

## Abstract

Metalation of the deprotonated dipyrrin (^AdF^L)Li with NiCl_2_(py)_2_ afforded the divalent Ni product (^AdF^L)NiCl(py)_2_ (**1**) (^AdF^L: 1,9-di(1-adamantyl)-5-perfluorophenyldipyrrin; py: pyridine). To generate a reactive synthon on which to explore oxidative group transfer, we used potassium graphite to reduce **1**, affording the monovalent Ni synthon (^AdF^L)Ni(py) (**2**) and concomitant production of a stoichiometric equivalent of KCl and pyridine. Slow addition of mesityl- or 1-adamantylazide in benzene to **2** afforded the oxidized Ni complexes (^AdF^L)Ni(NMes) (**3**) and (^AdF^L)Ni(NAd) (**4**), respectively. Both **3** and **4** were characterized by multinuclear NMR, EPR, magnetometry, single-crystal X-ray crystallography, theoretical calculations, and X-ray absorption spectroscopies to provide a detailed electronic structure picture of the nitrenoid adducts. X-ray absorption near edge spectroscopy (XANES) on the Ni reveals higher energy Ni 1s → 3d transitions (**3**: 8333.2 eV; **4**: 8333.4 eV) than Ni^I^ or unambiguous Ni^II^ analogues. N K-edge X-ray absorption spectroscopy performed on **3** and **4** reveals a common low-energy absorption present only for **3** and **4** (395.4 eV) that was assigned *via* TDDFT as an N 1s promotion into a predominantly N-localized, singly occupied orbital, akin to metal-supported iminyl complexes reported for iron. On the continuum of imido (*i.e.*, NR^2−^) to iminyl (*i.e.*, ^2^NR^−^) formulations, the complexes are best described as Ni^II^-bound iminyl species given the N K-edge and TDDFT results. Given the open-shell configuration (*S* = 1/2) of the iminyl adducts, we then examined their propensity to undergo nitrenoid-group transfer to organic substrates. The adamantyl complex **4** readily consumes 1,4-cyclohexadiene (CHD) *via* H-atom abstraction to afford the amide (^AdF^L)Ni(NHAd) (**5**), whereas no reaction was observed upon treatment of the mesityl variant **3** with excess amount of CHD over 3 hours. Toluene can be functionalized by **4** at room temperature, exclusively affording the *N*-1-adamantyl-benzylidene (**6**). Slow addition of the organoazide substrate (4-azidobutyl)benzene (**7**) with **2** exclusively forms 4-phenylbutanenitrile (**8**) as opposed to an intramolecular cyclized pyrrolidine, resulting from facile β-H elimination outcompeting H-atom abstraction from the benzylic position, followed by rapid H_2_-elimination from the intermediate Ni hydride ketimide intermediate.

## Abstract 2

Nickel-supported nitrenoids exhibit iminyl character, as determined by multi-edge XAS and TDDFT analysis, demonstrate efficacy for C–H activation and nitrene transfer chemistry.

## Graphical abstract

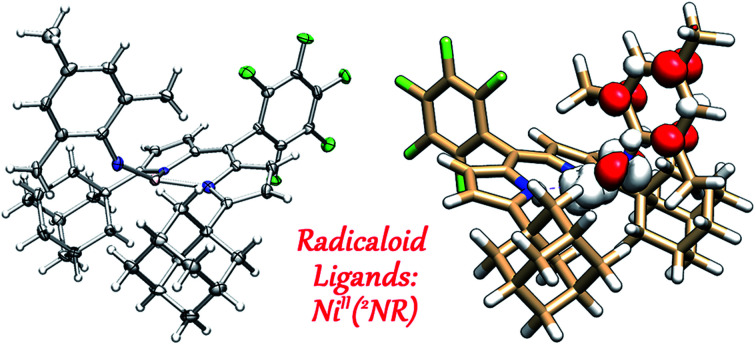

## 1. Introduction

Late 3d transition metal complexes bearing metal–ligand multiple bonds (MLMBs) have been intensely scrutinized as potential reagents to deliver the multiply-bonded functionalities to organic substrates.^1,2,4,5,8–43^ One method to endow the metal–ligand multiply bonded species with the capacity to undergo functional group transfer is to attenuate the metal–ligand bond order. We and others have pursued the synthesis of late transition metal, low-coordinate MLMBs to investigate their resulting group transfer reactivity.^1–6,8,9,14–18,35–43^ Our group has focused on the generation of highly electrophilic MLMBs by employing weak-field dipyrrin ancillary ligands.^37,39,40,42,44^ Ferric imido complexes bearing the dipyrrin ligand feature high-spin (*S* = 5/2) electronic structures,^37–40^ while the Co^III^ analogues thermally access open-shell excited states.^41,42^ The open-shell electronic structures for the Fe^37–40^ and Co^41,42^ complexes provide access to reactivity along the MLMB vector, where both inter- and intramolecular amination transfer to C–H bond substrates were observed. In both cases, however, the MLMBs were best formulated as imido functionalities. Oxidation of the Fe^III^ imidos elicits the formation of an open-shell iminyl, where oxidation is borne out at the NR functionality, not the metal center, leading to faster oxidative group transfer than their high spin imido analogues.^39,40^

**Fig. 1:**
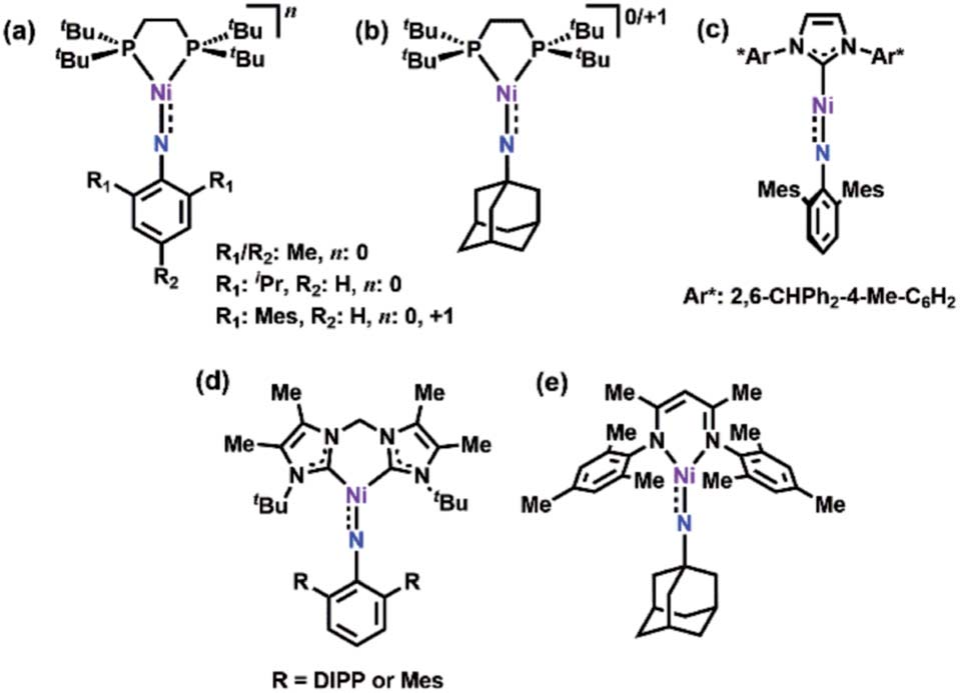
All previously reported Ni imides.^1–6^

Formation of later transition metal MLMBs can also diminish MLMB bond order in two ways: (1) higher d-electron counts necessarily populate more M–L antibonding orbitals; (2) progression through the 3d transition metal series should lead to diminished energetic overlap between the transition metal and N valence orbitals, potentially impacting the electronic structure and reactivity of the resultant MLMB.^17^ Indeed, four Ni imido systems have been thus far reported by Hillhouse^1,3–6,10–12^ and Warren.^2,13^ The seminal work by Hillhouse and coworkers first described the synthesis and reactivity of phosphine-supported Ni^II^ imidos (Fig. 1a and b),^10–12^ later establishing both higher valent imido cations (Fig. 1a and b)^4^ and lower-coordinate, carbene supported imido complexes (Fig. 1c and d).^3,5,6^ Warren and coworkers reported β-diketiminate supported neutral, nominally Ni^III^ imides (Fig. 1e).^2,13^ Of note, Hillhouse's two-coordinate, carbene supported Ni^II^ imido^5^ and Warren's Ni^III^ imido complexes^2,13^ are competent for nitrene transfer into vinylic and benzylic C–H bonds, respectively. Each example represents a highly electrophilic imido complex capable of functional group transfer. Given the promising capability Ni imido species exhibit towards C–H bond functionalization, we were interested in investigating the synthesis of dipyrrin-supported Ni analogues. We report herein the synthesis, characterization, and reactivity of two Ni iminyl complexes bearing aryl and alkyl *N*-substituents.

## 2. Results and discussion

**Fig. 2:**
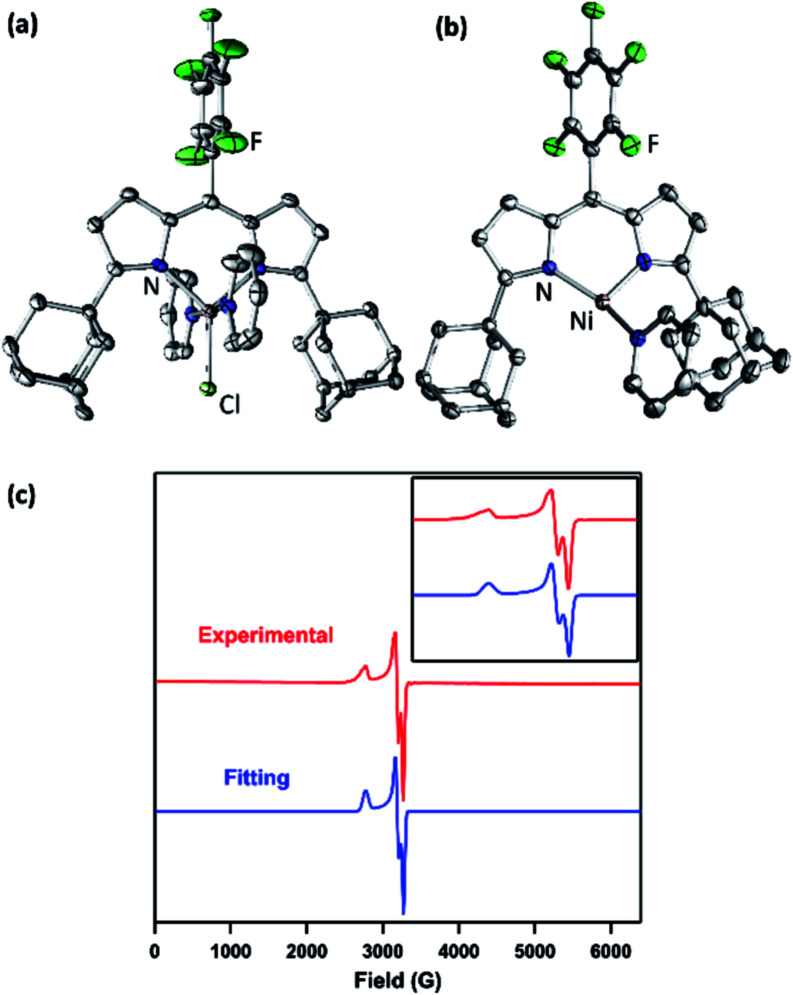
Solid-state molecular structure for (a) (^AdF^L)NiCl(py)_2_ (**1**) and (b) (^AdF^L)Ni(py) (**2**) with thermal ellipsoids at 50% probability level. Color scheme: Ni, pink; N, blue; C, gray; Cl, yellowgreen; F, green. H atoms omitted for clarity. (c) Frozen solution EPR spectrum of (^AdF^L)Ni(py) (**2**) collected at 77 K in toluene (red). Blue line represents a fit of the data using the program EasySpin.^7^ Fitting parameters: *S* = 1/2, *g*_1_ = 2.438, *g*_2_ = 2.121, *g*_3_ = 2.064.

**Scheme 1:**
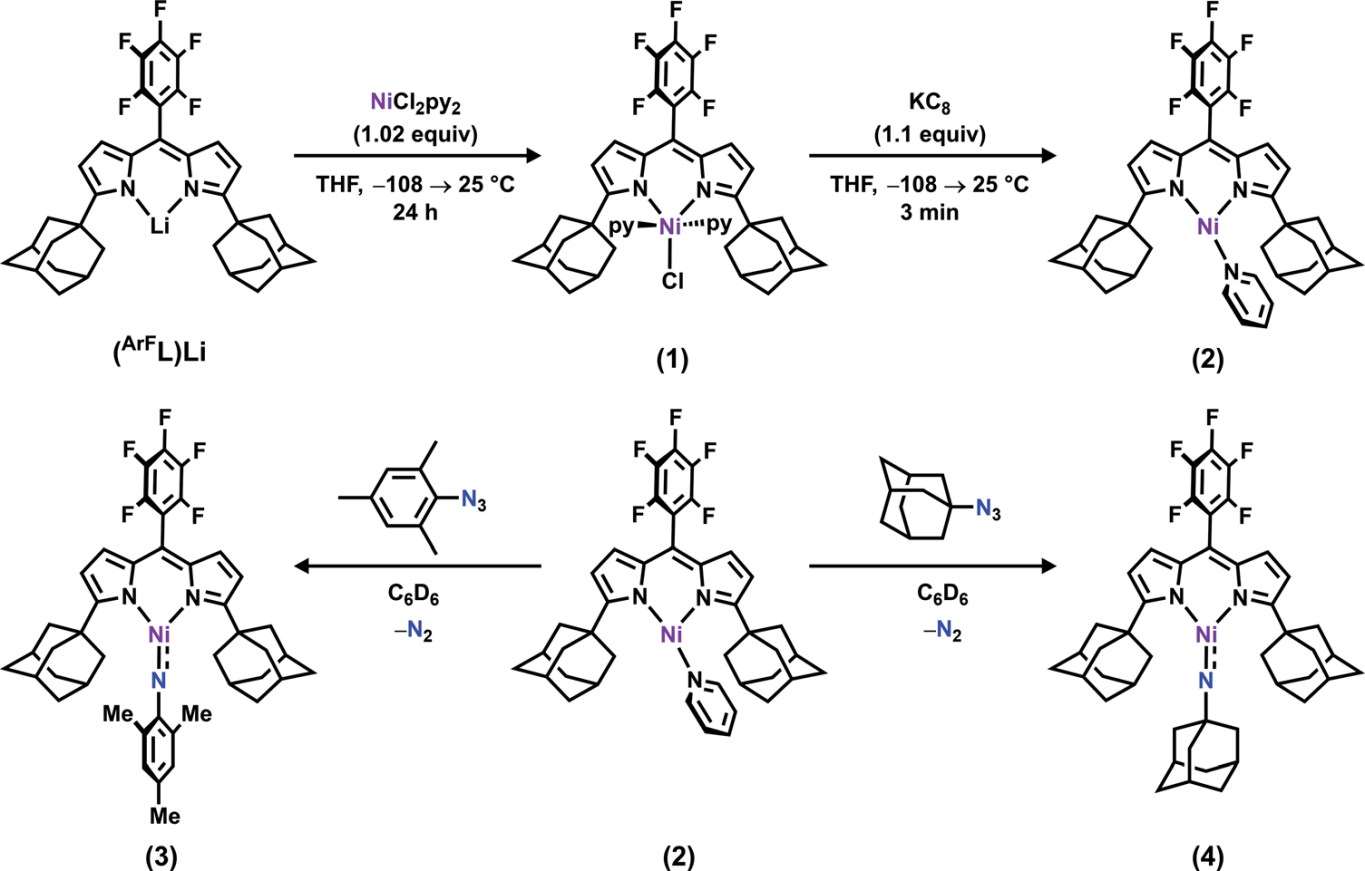
Synthesis of (^AdF^L)NiCl(py)_2_ (**1**), (^AdF^L)Ni(py) (**2**), (^AdF^L)Ni(NMes) (**3**), and (^AdF^L)Ni(NAd) (**4**).

The synthesis of a modified adamantyl-substituted dipyrrin scaffold proceeded cleanly *via* substitution of pentafluorobenzaldehyde for 2-(dimethoxymethyl)-1,3,5-trimethylbenzene in the original procedure.^37^ The fluorinated aryl *meso*-substituent on the dipyrrin anodically shifts the bound transition metal and provides a useful ^19^F handle for ^19^F NMR characterization.^45^ Metalation of (^AdF^L)H with Ni followed previously reported protocols for preparing Fe and Co congeners.^37,42,44^ Lithiation of (^AdF^L)H is carried out by adding PhLi as a solid to a frozen benzene solution of (^AdF^L)H.^37,44^ The addition of resultant (^AdF^L)Li in THF to a frozen solution of NiCl_2_(py)_2_ in THF afforded a dark-red, paramagnetic product. Crystals of the product were obtained by storing a concentrated solution of the material in hexanes at −35 °C. Single-crystal X-ray diffraction analysis revealed the product as the trigonal bipyramidal, bis-pyridine complex (^AdF^L)NiCl(py)_2_ (**1**, Fig. 2a), which exhibits a paramagnetically-shifted ^1^H NMR spectrum, suggesting a triplet ground state at room temperature (Scheme 1).

Chemical reduction of **1** with KC_8_ in thawing THF solution cleanly generated the pyridine adduct (^AdF^L)Ni(py) (**2**, Scheme 1) as a dark brown solid. Crystals of **2** suitable for single-crystal X-ray diffraction were obtained by storing a concentrated hexanes solution at −35 °C overnight (Fig. 2b). The solid-state structure of **2** unveils a pyramidally distorted T-shape geometry around the Ni center with an N_L_–Ni–N_py_ angle of 146.4(2)°, similar to the previously reported three-coordinate Ni^I^ β-diketiminate complex by Warren and coworkers (Fig. 1e).^2^ The EPR spectrum of **2** collected at 77 K in toluene indicates that the unpaired electron occupies a rhombic environment (*g*_1_ = 2.438, *g*_2_ = 2.121, *g*_3_ = 2.064; Fig. 2c).

### 2.1 Isolation and structural characterization of terminal nitrenoid complexes

**Fig. 3:**
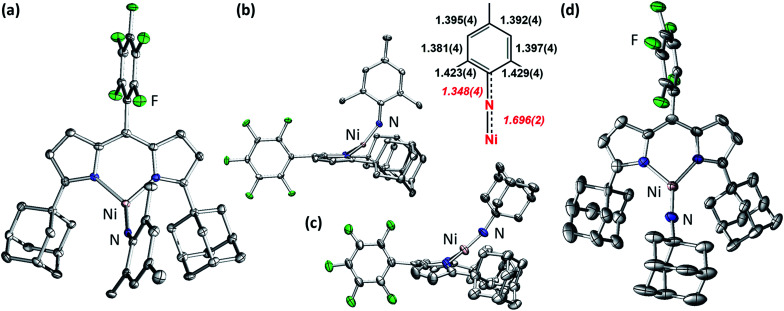
Solid-state molecular structures for the top-view (a) and side-view and aryl moiety bond metrics (b) of (^AdF^L)Ni(NMes) (**3**); the side-view (c) and top-view (d) of (^AdF^L)Ni(NAd) (**4**) with thermal ellipsoids at 50% probability level. Color scheme: Ni, pink; N, blue; C, gray; F, green. H atoms, solvent molecules, and positional disorder in **4** are omitted for clarity.

The addition of 1 equiv. of either MesN_3_ or AdN_3_ to a benzene solution of **2** resulted in instantaneous color change from dark brown to dark pink accompanied by vigorous effervescence due to N_2_ release from the azide (Scheme 1). The ^19^F NMR spectra of the crude reaction mixtures showed total consumption of **2** with clean conversion to new paramagnetic products. Single crystals of the respective reaction products were obtained by storing concentrated hexanes solutions at −35 °C overnight. The solid-state structures of the reaction products (Fig. 3a and d) confirmed N_2_ extrusion from the organoazides to install terminal NMes and NAd functionalities onto Ni in (^AdF^L)Ni(NMes) (**3**) and (^AdF^L)Ni(NAd) (**4**), respectively.

The Ni–N_im_ distance of 1.696(2) Å in **3** is longer than the bis-phosphine supported cationic Ni^III^ aryl imide [(dtbpe)Ni(NAr)]^+^ (1.674(3) Å)^4^ but similar to those three coordinated Ni^II^ aryl imides supported by the same ligand (1.697(2) → 1.703(4) Å)^1,3,4^ and bis-N-heterocylic carbene (NHC) ligand ((NHC_2_)Ni(NAr): 1.718(2) → 1.732(4) Å) (Fig. 1 and S7†).^6^ The imido linkage in **3** strongly deviates from linearity [∠(Ni–N_im_–C_Mes_): 146.5(2)°] in contrast to its linear bisphosphine supported analogue [(dtbpe)Ni(NAr)]^+^ [∠(Ni–N_im_–C_Ar_): 178.4(3)°]^4^ but similar to the bis-NHC system [(NHC_2_)Ni(NAr), ∠(Ni–N_im_–C_Ar_), Ar: 127.3(3)°, Mes; 170.0(2)°, DIPP] (Fig. 1 and S7†).^6^ The deviation from linearity for the Ni–N–C_Ar_ linkages occur most often with late transition metals featuring population of high-energy, antibonding (M–N)π* orbitals. Other factors that can contribute to distortions away from non-linearity include imido-group constraints incorporated into a chelate ring and crystal-packing effects within the crystalline lattice.^6^

Close inspection of the C–C bond lengths within the mesityl group of **3** (Fig. 3a and b) reveals the elongation of the C_*ipso*_–C_*ortho*_ bonds (1.423(4), 1.429(4) Å), analogous to our previously reported Fe^III^ iminyl species^37–40^ as well as all the previously reported Ni aryl imido complexes (Fig. S7†).^1,3–6^ The N_imido_–C_Mes_ distance in **3** (1.348(4) Å) is in the range of a partial C–N double bond, indicating delocalization of electron density from Ni–N_imido_ π system to the mesityl aryl moiety.^17^

The Ni–N_im_ distance of 1.642(7) Å in **4** is consistent with reported three-coordinate Ni^III^ alkyl imide supported by β-diketiminate ligand (1.662(2) Å)^2^ and Ni alkyl imides [(dtbpe)Ni(NAd)]^*n*^ (*n*: +1, 1.673(2); *n*: 0, 1.657 (5) Å);^3,4^ and comparatively shorter than the Ni–aryl imido complexes (Fig. 1 and S7†). The shortened Ni–N_im_ bond is consistent across all ancillary ligand platforms, potentially due to the lack of π-delocalization into the imido aryl moiety. The alkyl imido linkage in **4** is more linear [∠(Ni–N_im_–C_Ad_): 164.8(17)°] compared to **3**, and similar to all three reported Ni alkyl imides [∠(Ni–N_im_–C_Ad_): 163.0(2) → 165.2(4)°], regardless of ancillary ligand or oxidation state.^2–4^

### 2.2 Spectroscopic characterization of terminal nitrenoid complexes **3** and **4**

**Fig. 4:**
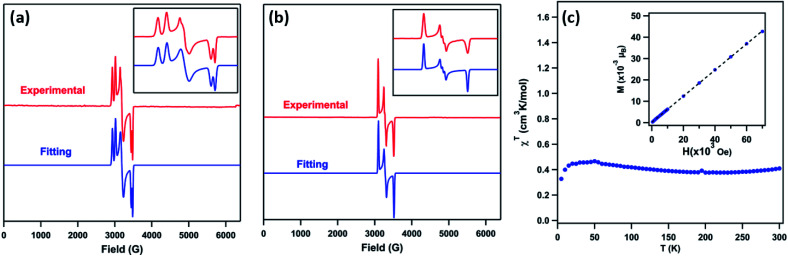
(a) Frozen solution EPR spectrum of (^AdF^L)Ni(NMes) (**3**) collected at 77 K: *S*_1_ = 1/2, *g*_11_ = 2.238, *g*_12_ = 2.106, *g*_13_ = 1.940; *S*_2_ = 1/2, *g*_21_ = 2.302, *g*_22_ = 2.128, *g*_23_ = 1.962, relative weight = 1 : 1 (red); blue line represents a fit of the data using the program EasySpin;^7^ (inset) enlarged spectrum showing hyperfine splitting attributable to two mesityl ring orientations. (b) Frozen solution EPR spectrum of (^AdF^L)Ni(NAd) (**4**) collected at 77 K: *S* = 1/2, *g*_1_ = 2.185, *g*_2_ = 2.063, *g*_3_ = 1.924 (red); blue line represents a fit of the data using the program EasySpin;^7,8^ (inset) enlarged spectrum showing hyperfine splitting, *A* = 21.3 G (^14^N, *I* = 1).^7,9^ (c) Variable-temperature susceptibility data for of **3** collected at 1.0 T, with *χ*_M_*T* = 0.41 cm^3^ K mol^−1^ at 295 K; (inset) solid-state magnetometry data for **3** of M *vs.* H at 100 K, showing the absence of ferromagnetic impurities.

The EPR spectrum of **3** (toluene, 77 K, Fig. 4a) displays two sets of rhombic, *S* = 1/2 signals (*g*_11_ = 2.238, *g*_12_ = 2.106, *g*_13_ = 1.940; *g*_21_ = 2.302, *g*_22_ = 2.128, *g*_23_ = 1.962), possibly due to two conformations of the mesityl group related by rotation, forming π-bonds with different metal based d-orbitals.^1,3,12^ The solid-state magnetometry measurement (Fig. 4c) confirmed that **3** remains low-spin (*S* = 1/2) up to 300 K. The EPR spectrum of **4** (toluene, 77 K, Fig. 4b) reveals a single rhombic absorption, supporting an *S* = 1/2 formulation (*g*_1_ = 2.185, *g*_2_ = 2.063, *g*_3_ = 1.924). The signal at *g*_2_ = 2.063 is split into a triplet with a hyperfine splitting constant of *A*(^14^N, *I* = 1) = 21.3 G. Similar spectroscopic features were observed in the Ni^III^ β-diketiminate imides with a hyperfine splitting constant of *A*(^14^N, *I* = 1) = 22 G.^2^ The *S* = 1/2 low-spin ground states for **4** were further verified by SQUID magnetometry (Fig. S5†) up to 300 K, in contrast to the Ni^III^ imide [(dtbpe)Ni(NAr)]^+^, which exhibits spin transition to high-spin *S* = 3/2 excited state.^4^

### 2.3 Redox stability of complex **3**

**Fig. 5:**
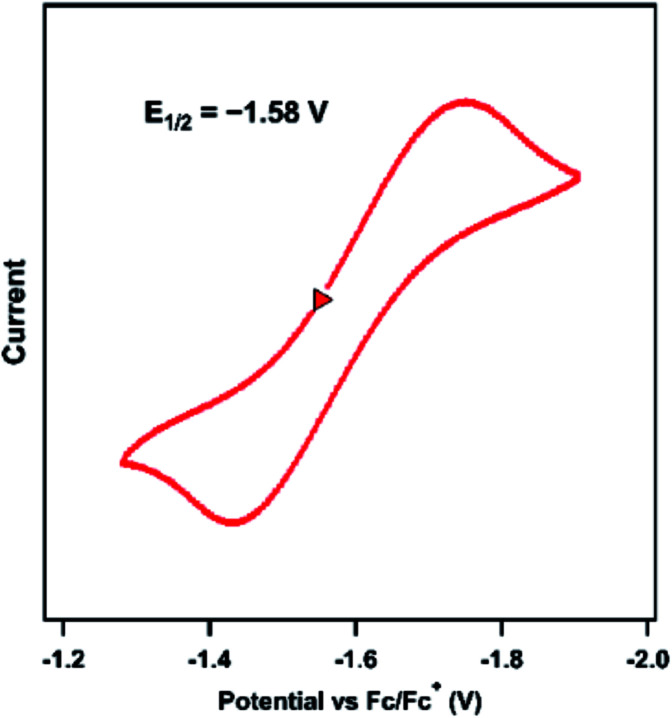
Cyclic voltammogram of **3** obtained in THF at 25 °C, with 0.1 M (^*n*^Bu_4_N)(PF_6_); 100 mV s^−1^; referenced to [Cp_2_Fe]^+/0^ couple; OCP = −1.25 V; Δ*E* = 310 mV.

The previously reported Ni imido complexes of the type [(dtbpe)Ni(NAr)]^+/0^ were accessible in both nominally Ni^II^ and Ni^III^ oxidation levels (Fig. 1a and b).^4^ Cyclic voltammetry (CV) was performed to assess the redox stability of the dipyrrin aryl imido analogue **3** (Fig. 5). The CV examination of **3** in THF shows a quasi-reversible reduction event at −1.58 V *versus* [Cp_2_Fe]^+/0^, cathodically shifted from the same formally Ni^II^/Ni^III^ couple of the related bis-phosphine supported Ni imide (*E*_1/2_ = −0.76 V, *vs.* [Cp_2_Fe]^+/0^, THF).^4^

The 0.8 V cathodic shift comparing [(dtbpe)Ni(NAr)]^+^*versus* neutral **3** likely largely reflects the change in molecular charge between the two species as opposed to effects originating with ligand field strength. Chemical reduction of **3** was attempted using decamethylcobaltacene 
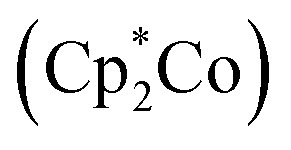
 or KC_8_ as a reductant in frozen-thawing THF.^46^ In both cases, unfortunately, only inseparable mixtures of decomposition of **3** as ascertained by analysis of the crude reaction mixture by ^19^F NMR spectroscopy were obtained. Similar results were obtained when similar chemical reduction was attempted with alkyl imide **4**.

### 2.4 Ni and N K-edge X-ray absorption near edge spectroscopies of complexes **3** and **4**

**Fig. 6:**
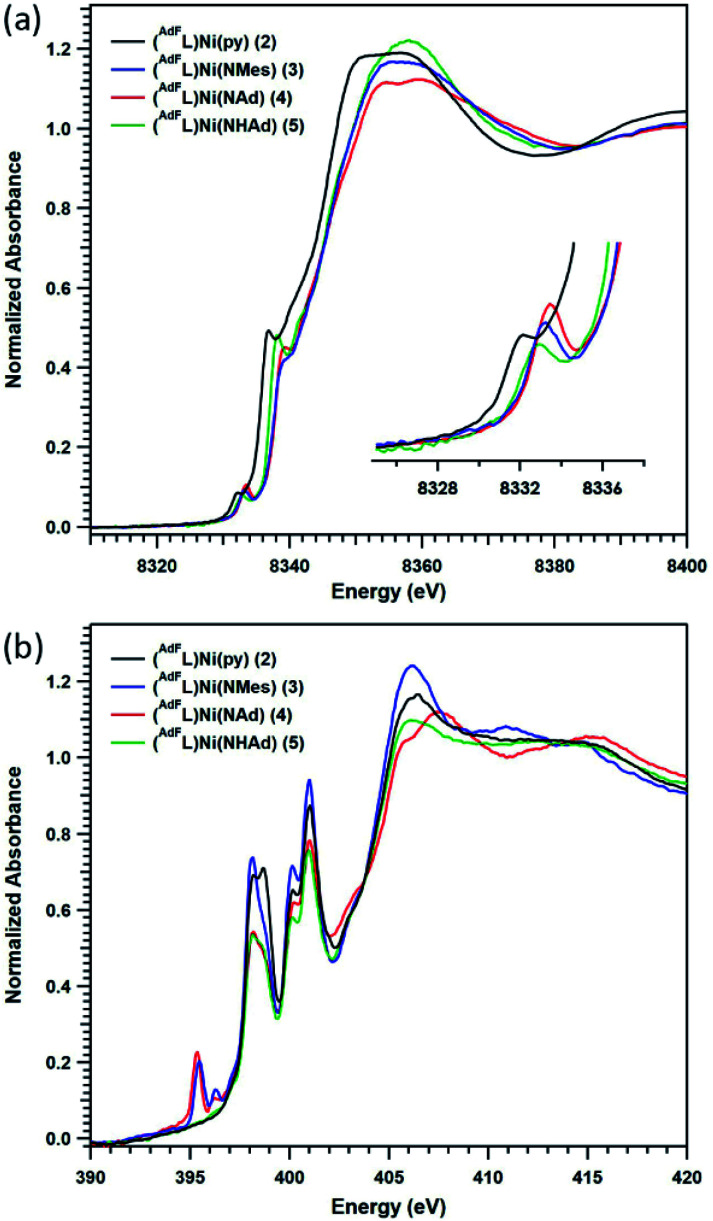
X-ray absorption spectroscopy: (a) Ni K-edge and (b) N K-edge absorption spectra of (^AdF^L)Ni(py) (**2**) (black), (^AdF^L)Ni(NMes) (**3**) (blue), (^AdF^L)Ni(NAd) (**4**) (red), and (^AdF^L)Ni(NHAd) (**5**) (green).

Given the challenges associated with metal oxidation assignment based solely on structural metrics, we investigated the suite of Ni complexes reported herein by multi-edge X-ray absorption spectroscopy (XAS). Formally Ni^I^**2** and the formally Ni^II^ (^AdF^L)Ni(NHAd) (**5**) (*vide infra*) were chosen as oxidation state references due to their similar coordination environments to those of complexes **3** and **4**. The Ni K-edge (Fig. 6a) spectra reveal pre-edge absorption features for each of the complexes **2–5**, conventionally assigned to Ni 1s → 3d excitations. The maxima for the pre-edge features increase in energy across the series from Ni^I^**2** (8332.0 eV), to formally Ni^II^**5** (8332.8 eV), to the terminal MLMB complexes **3** (8333.0 eV) and **4** (8333.2 eV). The pre-edge maximum shifts 0.8 eV on going from Ni^I^**2** to Ni^II^**5**; while the terminal imidos reveal smaller shifts in pre-edge maxima from **5** (**3**, 0.2 eV; **4**, 0.4 eV). Comparing the aryl and alkyl imides (**3**, **4**), the pre-edge absorption feature of the alkyl species is shifted to higher energy relative to that of the aryl congener, likely resulting from conjugation of the aryl moiety in **3** to the singly occupied acceptor molecular orbital. A similar observation was made comparing aryl- and alkyl-substituted iminyl complexes on Fe^III^.^40^

To gain further insight into the redox states in the Ni/N pairs in **3** and **4**, the nitrogen valence was probed directly using nitrogen K-edge XAS. As seen in Fig. 6b, pre-edge features at 398.0 and 400.9 eV are observed for all the complexes surveyed (**2–5**) that can be assigned as transitions into high-lying antibonding molecular orbitals of N 2p parentage.^40,47^ Two low-lying pre-edge absorptions are present for both **3** and **4** at 395.4 eV, accompanied by a smaller side-band at 396.3 eV. These peak energies are consistent with those encountered for the redox-active N-donor ligands in Fe-iminyl (394.5 and 394.8 eV),^40^ Ni–aminyl (397.0 eV),^47^ and Cu–nitrene^48^ (doublet with peaks at 395.3 and 395.9 eV) complexes. This tentative assignment of these transitions as N 1s into partially occupied, N-localized orbitals present in **3** and **4**, respectively is further supported by time-dependent density functional theory (TDDFT) calculations (*vide infra*). However, quantitative covalency analysis was precluded by sensitivity of these samples to rapid photodamage that principally affected the low-energy pre-edge peaks of interest (Fig. S13†).

### 2.5 Theoretical analysis for the electronic structures of complexes **3** and **4**

DFT calculations were carried out to probe the electronic structures of **3** and **4**. Calculations were carried out on the experimental (crystallographic) structures using the BP86 ^49,50^ generalized gradient approximation (GGA) functional as well as the B3LYP^51,52^ hybrid density functional. The broken symmetry (BS)^53–55^ method was applied in both cases to ascertain whether antiferromagnetic coupling between a high-spin d^8^ Ni^II^ and a ligand radical (*i.e.*, imido oxidized to an iminyl) could plausibly be invoked to yield the experimentally observed *S* = 1/2 ground states. No BS(2,1) solution was obtained for either species using the BP86 functional. Analogous calculations using B3LYP gave broken symmetry solutions with corresponding orbital overlap values near unity (**3** = 0.68, **4** = 0.89) and whose total energies were effectively identical to those obtained using a standard unrestricted DFT approach.

**Fig. 7:**
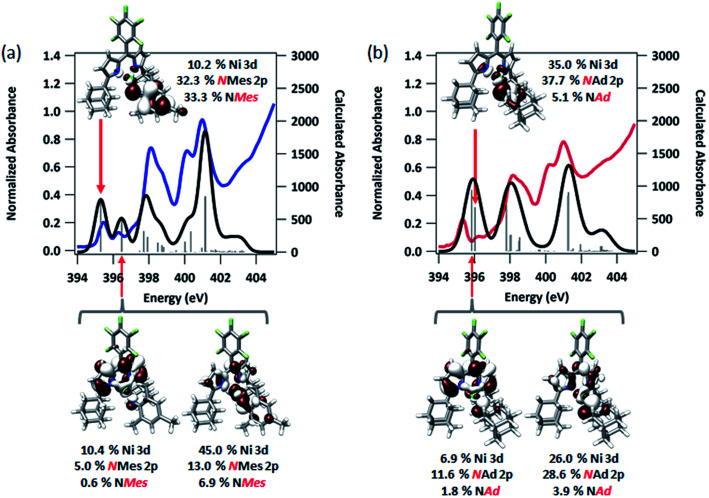
Experimental and TDDFT-calculated N K-edge XAS pre-edge regions for (a) (^AdF^L)Ni(NMes) (**3**), and (b) (^AdF^L)Ni(NAd) (**4**). Calculations were initiated from spin-unrestricted B3LYP single points using the CP(PPP) basis set on Ni and the ZORA-def2-TZVP(-f) basis set on all other atoms. Energies for the calculated spectra were adjusted using a linear correction presented as Fig. S15.† Unrestricted Kohn–Sham orbitals corresponding to the acceptor MOs involved the excitation giving the *ca.* 395 eV pre-edge peak are plotted at an isovalue of 0.03 au.

To afford some justification for selecting between BP86 and B3LYP results, TDDFT calculations^47,56,57^ to model the N K-edge XAS obtained for **3** and **4** were carried out starting from either unrestricted single point solution. Spectra calculated using BP86 gave insufficient resolved peak structure to reliably correlate to the experimental data (Fig. S14†), whereas B3LYP-calculated spectra adequately reproduced pre-edge features, with a linear fit of experimental to calculated data giving *R*^2^ = 0.99 (Fig. 7). Agreement between calculated and experimental spectra is quite good in the case of **3**, but less so in the case of **4**.

The first peak in the N K-edge XAS of **3** is predicted as an excitation from N 1s to an MO dominated by iminyl character, because the MO is less than 50% Ni in character.^58^ This MO is delocalized over the NMes fragment and is comprised of 32.3% N 2p, 33.3% Mes arene π*, and 10.2% Ni 3d. The weaker, second pre-edge peak is assigned as an excitation involving a near equal mix of two acceptor MOs featuring large (10–20%) contributions from the orthogonal N_im_ 2p orbitals as well as dipyrrin π* contributions. In the case of **4**, qualitatively similar excitations are involved, although the ordering is reversed from **3**: the first excitation features contributions from 2 MOs, while the second excitation is analogous to the first excitation of **3**. The estimated error in peak energy prediction from our prior N K-edge TDDFT study is on the order of 0.5 eV, so this discrepancy is not unexpected. Regardless, the pre-edge of **4** also features excitations to an MO with a large degree of N_im_ N 2p vacancy. Much less delocalization is possible in the imido fragment of **4**, consequently this acceptor MO features 42.8% total contribution from NAd (37.7% N 2p), and 35.0% Ni 3d.

**Fig. 8:**
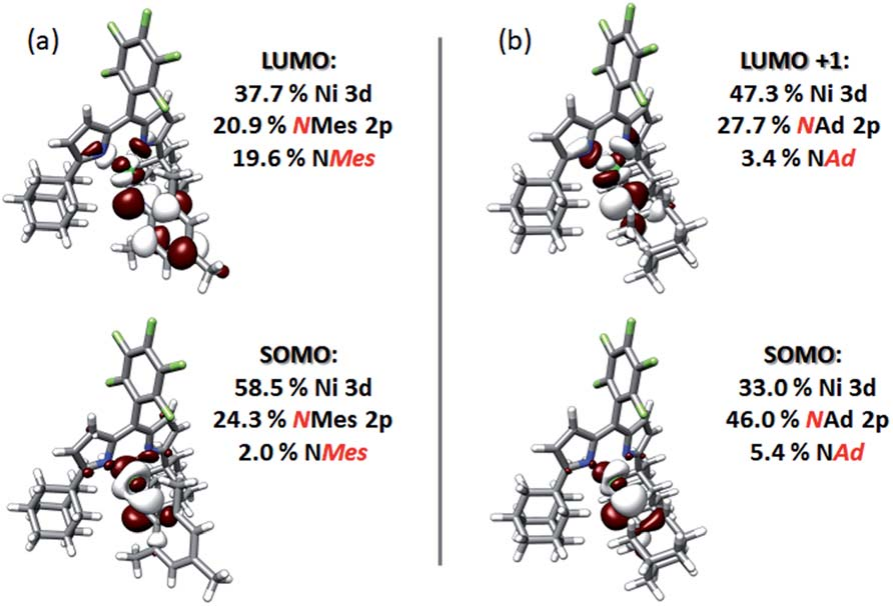
Frontier quasi-restricted orbitals (QROs) calculated for (a) (^AdF^L)Ni(NMes) (**3**), and (b) (^AdF^L)Ni(NAd) (**4**). QROs were generated from spin-unrestricted B3LYP orbitals produced using the CP(PPP) basis set on Ni and the ZORA-def2-TZVP(-f) basis set on all other atoms. Orbitals are plotted at an isovalue of 0.03 au.

Quasi-restricted orbitals (QROs)^59^ were generated from the unrestricted B3LYP solutions to facilitate interpretation of the ground-state electronic structures of **3** and **4** (Fig. 8). The picture that emerges for both is that the unpaired electron resides in a MO of principally Ni 3d and N_im_ character reflecting a π* interaction. For **3**, this MO comprises 58.5% Ni 3d and 24.3% NMes N 2p character. For **4**, corresponding parentages are 33.0% Ni 3d and 46% NAd N 2p. The *ca.* 2-fold larger N 2p contribution to the SOMO of **4** is consistent with the resolved ^14^N HFC in its EPR spectrum—such fine structure is not resolved in the spectrum of **3**. Each complex has one remaining unoccupied orbital featuring Ni 3d character, reflecting a π* interaction with N_im_ 2p. This is the LUMO for **3**, which comprises 37.7% Ni 3d and 20.9% NMes N 2p. For **4**, this is LUMO+1, which features 47.3% Ni 3d and 27.7% NAd N 2p. The ground state electronic configuration for **4** appears more consistent with a formal iminyl assignment, while the larger Ni parentage of the SOMO (58.5% Ni 3d) for **3** suggests **3** presents more imido-like character. Overall, the highly covalent Ni–N_im_ interactions can be viewed as total donation of a single electron from the N_im_ 2p orbitals involved in Ni–N bonding, giving rise to the iminyl character observed.

### 2.6 Reactivity of complexes **3** and **4**

**Scheme 2:**
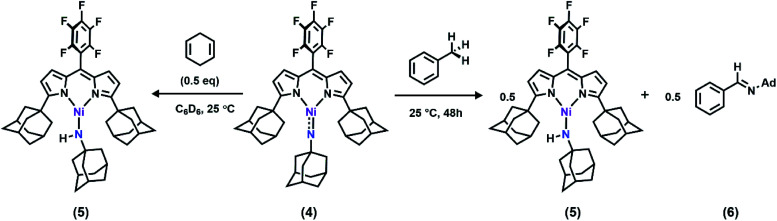
(Left) Reaction between **4** and 0.5 equiv. of CHD at 25 °C, quantitatively generating **5***via* facile H-atom abstraction; (right) reaction between **4** and toluene as solvent at 25 °C, generating **5** and **6***via* C–H functionalization followed by β-hydride elimination.

**Fig. 9:**
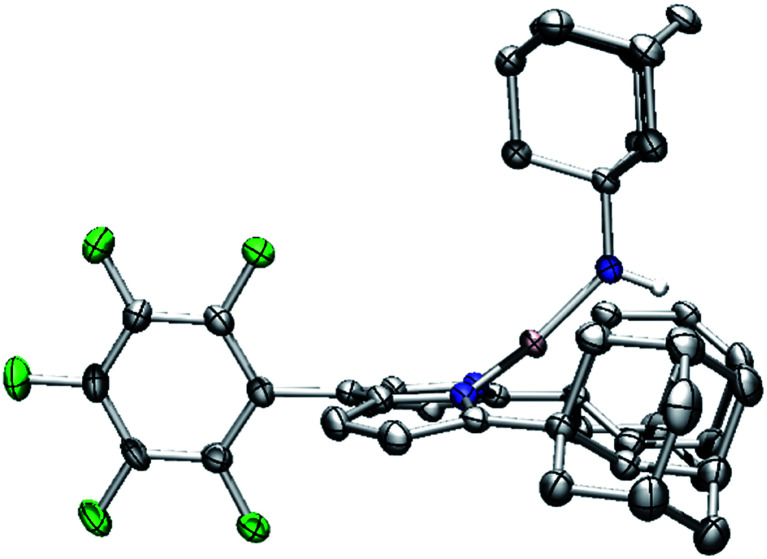
Molecular structure of (^AdF^L)Ni(NHAd) (**5**) with the ellipsoid probabilities set at 50%. C, gray; N, blue; H, white; Ni, pink; F green.

With the electronic structure of **3** and **4** described in detail, we next sought to explore their potency toward C–H amination and inspect the effects of substituents on the N_im_ in group transfer reactivity. Exposure of **4** to 0.5 equiv. of 1,4-cyclohexadiene (CHD) at room temperature resulted in instantaneous conversion into a new paramagnetic species as evidenced by the paramagnetically-shifted ^1^H and ^19^F NMR spectra (Scheme 2). Single crystals obtained from storing a concentrated hexanes solution of this reaction product at −35 °C overnight permitted structural determination by X-ray diffraction analysis. The product from reacting **4** with CHD is the corresponding amido species (^AdF^L)Ni(NHAd) (**5**, Fig. 9). The amide hydrogen atom was located in the difference map. Similar reactivity was observed in Ni^III^ imides supported by β-diketiminate reported by Warren and coworkers.^2,13^ Amide **5** was not stable at elevated temperature as complete decomposition of **5** into free ligand was observed at 40 °C in 6 hours. Interestingly, treatment of **3** with 100 equiv. of CHD resulted in no reaction over the course of 3 hours at room temperature, whereas decomposition was observed above 60 °C. A similar reactivity discrepancy was also observed in our previously reported Fe^III^ adamantyl and mesityl imido species, and could be explained by the lower radical density on N_im_ in **3** as well as steric-shielding of the electro-active orbital by the mesityl *ortho*-methyl units.^39,40^

**Fig. 10:**
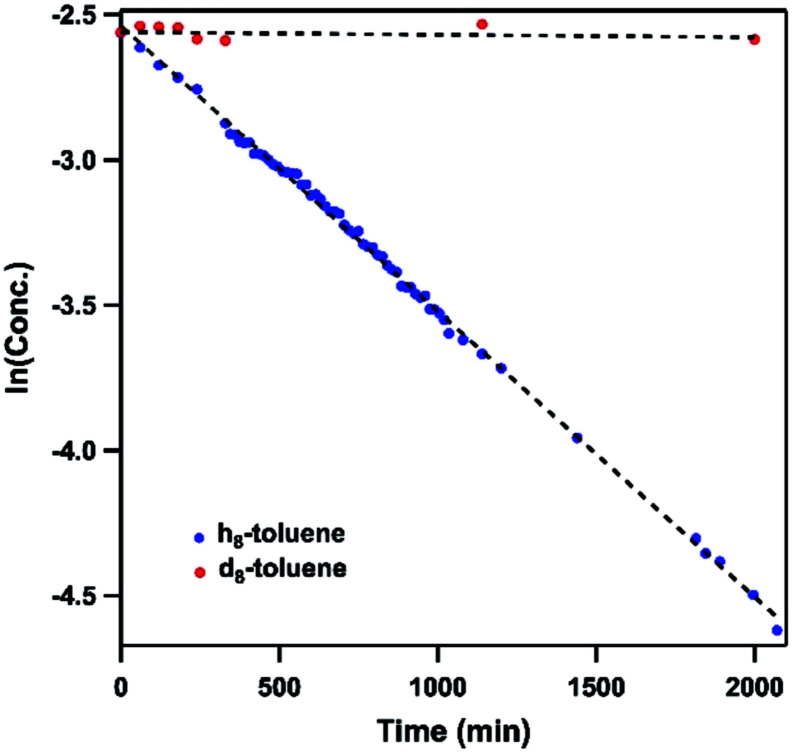
Concentration profile of **4** during reaction between (h_8_/d_8_-)toluene and **4** by ^19^F NMR spectroscopy. Trifluorotoluene is used as internal standard.

Dissolving **4** in toluene resulted in slow conversion into **5** along with decomposition into ligand over the course of two days. The amount of **5** generated is quantified by ^19^F NMR spectrum to be 46% against an internal standard (trifluorotoluene), and the organic product is identified to be *N*-1-adamantyl-benzilidene (**6**) as evidenced by ^1^H NMR spectrum of the quenched reaction mixture (Scheme 2). The concentration of **4** in toluene was monitored by ^19^F NMR spectroscopy and can be fitted as a first-order decay (Fig. 10). An intermolecular KIE of 40 was measured over 80 days when d_8_-toluene was used as the solvent. However, due to the slow rate of the reaction, decomposition of **4** can also contribute to the large KIE value. Based on the C–H amination reaction mediated by previously reported Ni^III^ β-diketiminate imides, we hypothesized that **4** can outcompete **5** (*in situ*) at capturing tolyl radical, giving rise to a secondary amido intermediate. This intermediate would then undergo β-hydride elimination and release the imine product. A similar mechanism was proposed by Warren *et al.* as a rationale towards their crystallographically characterized rearranged Ni^II^ product.^2,13^ An alternative mechanism would involve H-atom abstraction of benzyladamantylamine with a second equivalent of **4**, generating both the imine product observed and **5** as a byproduct.

**Scheme 3:**
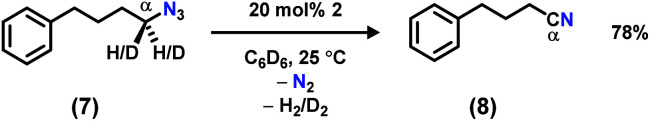
Catalytic conversion of **7** to **8** at 25 °C using 20 mol% of **2**.

In order to facilitate the radical recombination with the amido species formed *in situ*, the activated C–H bond was installed onto the azide. Addition of 1 equiv. of (4-azidobutyl)benzene (**7**)^22^ to a C_6_D_6_ solution of **2** resulted in instantaneous color change from dark brown to dark pink along with vigorous effervescence. The bubbling ceased after 10 min along with the solution reversion to a dark brown color, and ^19^F NMR spectrum revealed full regeneration of **2**. However, rather than the anticipated cyclized pyrrolidine product,^22,31,41^^1^H NMR analysis revealed the major organic product to be 4-phenylbutanenitrile (**8**, Scheme 3). To test the possibility of catalytically converting azide **7** to nitrile **8**, 3–6 equivalents of azide **7** were added slowly to a C_6_D_6_ solution of **2**. Full decomposition of catalyst **2** was observed when greater than five equivalents of the azide substrate **7** were used, potentially due to the high concentration of product nitrile **8**. With 20 mol% catalyst loading of **2**, azide **7** is catalytically converted to 4-phenylbutanenitrile **8**, providing a moderate isolated yield of 78%. We propose the product is formed by iminyl generation following N_2_ extrusion from azide **7**; β-H elimination (originating from the α-methylene unit) outcompetes intramolecular H-atom abstraction from the benzylic position to generate a Ni(hydride)(ketimide) intermediate. A rapid H_2_-elimination *via* deprotonation of the ketimide Ni(hydride) furnishes the final nitrile **8**. Attempts to slow down this pathway by *deutero*-substitution at the *gem* position were ineffective.

## 3. Conclusions

Both Ni^II^ aryl and alkyl nitrenoid adducts were prepared and characterized. A significant amount of spin density is delocalized on to the nitrenoid fragment as determined by SC-XRD, EPR spectroscopy, XAS, and quantum calculations. On the continuum between imido (*i.e.*, Ni^III^(NR)) and iminyl (*i.e.*, Ni^II^(^2^NR)) ligation, the alkyl nitrenoid **4** exhibits features consistent with an iminyl formulation whereas the singly occupied molecular orbital on **3** features more Ni character reminiscent of imido formulations. The nitrenoid bond distortions reported herein are similar to those observed in previously reported Ni^III^(NR), suggesting that the iminyl electronic structure proposed here may be applicable to those compounds as well.^2,4,13^ Indeed, the short Ni–NR bonds are commonly invoked for substantive metal–ligand multiple bond formation. However, the presence of low-energy absorption features in the N K-edge XAS data and corresponding assignments from TDDFT strongly evidence N 2p-localized vacancies. The C–H bond functionalization reactivities of these Ni(NR) species were closely examined. The Ni(NR) aryl congener is shown to be much less reactive than the alkyl variant, potentially owing to the greater spin concentration on Ni in the spin-bearing orbital. In the C–H bond amination reactions of Ni(NAd) with toluene, the corresponding imine was determined to be the major organic product, presumably due to recombination of the tolyl radical with the Ni(NAd) outcompeting that with the generated Ni(NHAd) amide. A subsequent β-hydride elimination would generate the secondary imine product. Future work will examine the efficacy of the (dipyrrin)Ni complexes to perform ring-closing C–H bond amination with substrates that cannot undergo α-hydride migration as observed here.

## Conflicts of interest

There are no conflicts to declare.

## Supplementary Material

SC-011-C9SC04879K-s001

SC-011-C9SC04879K-s002
