# Supplementary material for: Synthesis, characterization and C–H amination reactivity of nickel iminyl complexes
Source: Chem Sci. 2019 Dec 11;11(5):1260–8. doi: 10.1039/c9sc04879k (PMC8147896; doi:10.1039/c9sc04879k)
Supplement: SC-011-C9SC04879K-s001 [file SC-011-C9SC04879K-s001.pdf]

Electronic Supplementary Material (ESI) for Chemical Science.
This journal is © The Royal Society of Chemistry 2019

Synthesis, Characterization and C–H Amination Reactivity of Nickel Iminyl Complexes

Supporting Information

Yuyang Dong,† James T. Lukens,‡, Ryan M. Clarke,† Shao-Liang Zheng,† Kyle M. Lancaster,‡ and Theodore A Betley*,†

†Department of Chemistry and Chemical Biology, Harvard University, 12 Oxford Street, Cambridge, Massachusetts 02138, United States

‡Department of Chemistry and Chemical Biology, Baker Laboratory, Cornell University, Ithaca, New York 14853, United States

General Considerations 3
Characterization and Physical Measurements 4
XAS Measurements 5
Metal Complexes Synthesis 6
Experimental procedure for reaction between CHD and 3 11
Experimental procedure for reaction between CHD and 4 11
Experimental procedure for reaction between toluene and 4 12
Experimental procedure for concentration profile of 4 during reaction between 4 and toluene 12
Experimental procedure for reaction between CHD and 3 12
Experimental procedure for catalytic reaction between 7 and 20 mol% of 2 13
Figure S1. Frozen solution EPR spectrum of (^{AdF}L)Ni(py) (2) 14
Figure S2. Frozen solution EPR spectrum of (^{AdF}L)Ni(NMes) (3) 15
Figure S3. Frozen solution EPR spectrum of (^{AdF}L)Ni(NAd) (4) 16
Figure S4. Magnetization data of (^{AdF}L)Ni(NMes) (3) 17
Figure S5. Magnetization data of (^{AdF}L)Ni(NAd) (4) 18
Figure S6. Cyclic voltammogram of (^{AdF}L)Ni(NMes) (3) 19
Figure S7. Relevant bond metrics in all Ni Imides 20
Figure S8. ^{19}F NMR spectra of reaction between CHD and 3 at 25 °C 21
Figure S9. ^{19}F NMR spectra of reaction between CHD and 4 at 25 °C 22
Figure S10. ^{19}F NMR and ^{1}H NMR spectra of reactions between toluene and 4 at 25 °C ... 23
Figure S11. Concentration profile of reaction between toluene and 4 by ^{19}F NMR spectroscopy at 25 °C 25
Figure S12. ^{1}H, ^{13}C, and ^{19}F NMR spectra of reaction between 7 and 2 at 25 °C 26

Figure S13. Progressive N K-edge XAS scans obtained for 4 30
Figure S14. TD-DFT calculated N K-edge XAS for 3 and 4 31
Figure S15. Correlation of TD-DFT calculated N K pre-edge peak energies to experimental values 32
X-ray Diffraction Techniques 33
Table S1. X-ray Diffraction Experimental Details 35
Figure S16. Solid-state structure of (^{AdF}L)Ni(py) (1) 37
Figure S17. Solid-state structure of (^{AdF}L)Ni(py) (2) 38
Figure S18. Solid-state structure of (^{AdF}L)Ni(NMes) (3) 39
Figure S19. Solid-state structure of (^{AdF}L)Ni(NAd) (4) 40
Figure S20. Solid-state structure of (^{AdF}L)Ni(NHAd) (5) 41
References 42

General Considerations

All manipulations of metal complexes were carried out in the absence of water and dioxygen using standard Schlenk techniques, or in an MBraun inert atmosphere drybox under a dinitrogen atmosphere. All glassware was oven dried overnight and cooled in an evacuated antechamber prior to use in the drybox. Benzene, hexanes, toluene, and tetrahydrofuran were dried over 4 Å molecular sieves (Strem) prior to use. Chloroform-*d* was purchased from Cambridge Isotope Labs and used as received. Benzene-d_6 was purchased from Cambridge Isotope Labs and was degassed and stored over 4 Å molecular sieves prior to use. Compounds 2,4,6-trimethylaniline, sodium azide, sodium nitrite, 1-azoadamantane, graphite, and potassium were purchased from Aldrich. Compounds 2,4,6-trimethylphenyl azide, potassium graphite and bis(pyridine)nickel(II) dichloride were prepared according to published procedures.[1-2] Celite® 545 (*J.* T. Baker) was dried in a Schlenk flask for 24 h under dynamic vacuum while heating to at least 150 °C prior to use in a drybox. Silica gel 32-63 μ (AIC, Framingham, MA) was used as received.

Characterization and Physical Measurements

^{1}H, ^{13}C, ^{19}F and were recorded on Varian Unity/Inova 500 MHz- spectrometers. ^{1}H and ^{13}C NMR chemical shifts are reported relative to $SiMe_4$ using the chemical shift of residual solvent peaks as reference. ^{19}F NMR chemical shifts are reported relative to an external standard of boron trifluoride diethyl etherate.

Elemental analysis was carried out using PerkinElmer 2400 CHNS/O Series II System.

EPR spectra were obtained on a Bruker EleXsys E-500 CW-EPR spectrometer. Spectra were measured as frozen toluene or 2-methyltetrahydrofuran glasses at a microwave power of 0.6325–2 mW. Spectral simulations and fitting incorporating spin state and rhombicity were performed using VisualRhombo and EasySpin.[3]

Magnetic data were collected using a Quantum Design MPMS-5S SQUID magnetometer. Measurements were obtained for finely ground microcrystalline powders restrained in a frozen eicosane matrix within polycarbonate capsules. Samples were prepared under a dry nitrogen atmosphere by packing the powder in a gelcap and adding warm liquid eicosane, which formed a solid wax upon cooling. Dc susceptibility measurements were collected in the temperature range 5–300 K under a dc field of 1000, 5000 or 10000 Oe. Dc magnetization measurements were obtained in the temperature range 1.8–10 K under dc fields of 1, 2, 3, 4, 5, 6, and 7 T. The susceptibility data was corrected for contributions from the sample holder and eicosane, as well as the core diamagnetism of the sample using Pascal's constants.

$$\chi_m = \frac{\chi M}{mH} \tag{S.1}$$

Molar susceptibilities were calculated from (χ_m) by converting the calculated magnetic susceptibility (χ) obtained from the magnetometer according to Eq. S.1.

XAS Measurements

All data were measured at the Stanford Synchrotron Radiation Lightsource (SSRL) under ring conditions of 3.0 GeV and 500 mA. All samples were prepared in an inert-atmosphere glovebox and were measured as solids. For Ni K-edge measurements, samples were ground with BN to a final concentration of 10 weight % Ni, pressed into 1 mm aluminum spacers and sealed with 37 μm Kapton tape. For N K-edge measurements, samples were ground to a fine powder and spread in a thin layer on carbon tape affixed to an Al sample rod.

Ni K-edge measurements were carried out at SSRL Beamline 7-3. Samples were maintained at 10 K in a liquid He cryostat during data collection. Spectra were collected in transmission mode, with X-rays detected by ionization chambers immediately downstream and upstream of the sample. A Ni foil and a third ionization chamber upstream of the sample were used for internal energy calibration, setting the first inflection point of the Ni foil scan to 8331.6 eV. Data were collected from 8010.0 to 8371.0 eV. Four scans of each sample were collected and averaged. Spectra were processed using SIXPACK[4] and Igor Pro 6.37. The region below 8300 eV was used to fit a linear background, while the post-edge was flattened with a piecewise spline and set to an average intensity of 1.

N K-edge XAS measurements were collected on the 31-pole wiggler beam line 10-1 with a 600 lines/mm spherical grating monochromator and 20 μm entrance and exit slits. Data were measured by monitoring the change in sample current through detection of the total electron yield (TEY). The drain current was normalized to incident photon flux with a gold-grid reference monitor. Incident beam energy was calibrated by comparison of the Ni L_3 second order transition at 426.35 eV in a reference sample placed upstream of the sample chamber. Samples were maintained at room temperature under an ultra-high vacuum (10^{-9} Torr) during collection. Data were collected from 380.0 to 450.0 eV. Seven scans were measured and averaged for each compound. Processing was carried out using PyMCA. Background subtraction was achieved by fitting a line to the pre-edge region below 395.0 eV and subtracting from the entire spectrum. The post edge region above 410.0 eV was fit to a flattened polynomial and normalized to 1.0.

Metal Complexes Synthesis

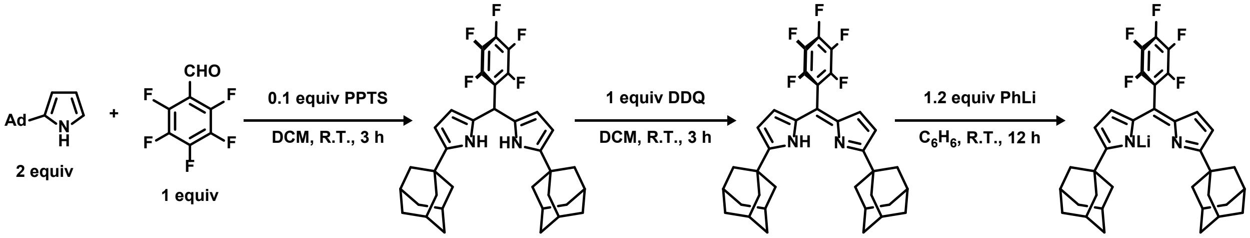

1,9-di(1-adamantyl)-5-perflorophenyldipyrromethane: To a stirring solution of pentafluorobenzaldehyde (4.90 g, 25.0 mmol, 1.00 equiv) in 500 mL of dichloromethane was added 2-adamantyl pyrrole (10.1 g, 50.0 mmol, 2.00 equiv) followed by pyridinium *p*-toluene sulfonate (1.26 g, 5.00 mmol, 0.100 equiv). The mixture was stirred at room temperature. After 3 h, the mixture was passed through a silica plug (300 cm^3) eluting with dichloromethane. The filtrate was concentrated in vacuo to afford 1,9-di(1-adamantyl)-5-perflorophenyldipyrromethane as a yellow solid (14.2 g, 98%). The product was carried onto the next step without any further purifications.

(^{AdF}L)H: To a stirring solution of 1,9-di(1-adamantyl)-5-perflorophenyldipyrromethane (14.2 g, 24.5 mmol, 1.00 equiv) in 300 mL of dichloromethane, 2,3-dichloro-5,6-dicyanoquinone (DDQ) (5.56 g, 24.5 mmol, 1.00 equiv) was added in one portion to give a dark brown solution. After stirring for 12 h, the solution was concentrated in vacuo. The resulting residue was extracted using benzene, and the solution was filtered through a silica plug (300 cm^3) to give a dark yellow filtrate. The volatiles were removed in vacuo to give 1,9-di(1-adamantyl)-5-perflorophenyldipyrromethene (^{AdF}L)H as a yellow solid (13.5 g, 95%). ^{1}H NMR (500 MHz, C_6D_6): δ ppm 13.50 (br. s, 1H), 6.37 (d, J = 4.3 Hz, 2H), 6.25 (dd, J = 4.2, 0.6 Hz, 2H), 2.08–1.91 (m, 18H), 1.77–1.61 (m, 12H); ^{13}C NMR (125 MHz, C_6D_6): δ ppm 170.78, 148.04 (d, J = 123.8 Hz), 144.31 (d, J = 127.5 Hz), 142.27, 140.44 (d, J = 123.12 Hz), 129.72, 124.24, 117.82, 115.06, 44.87, 39.86, 38.62, 31.68; ^{19}F NMR (470 MHz, C_6D_6): δ ppm −139.02–−139.20 (m, 2F), −153.32 (t, J = 21.6 Hz, 1F), −160.85–−161.91 (m, 2F). HRMS (ESI$^+$, m/z for $[M+H]^+$) Calc for $[C_{35}H_{35}F_5N_2+H]^+$: 579.2793, found: 579.2766.

(^{AdF}L)Li: In a 200 mL round-bottomed flask, (^{AdF}L)H (13.5 g, 23.3 mmol, 1.00 equiv) was dissolved in 100 mL of benzene and frozen. Phenyl lithium (2.35 g, 28.0 mmol, 1.20 equiv) was then added as a solid. The mixture was warmed up to room temperature and stirred overnight. After 12 h, the dark orange mixture was filtered through a Celite plug (300 cm^3) to afford an orange

solution. The volatiles were removed in vacuo to give (^{AdF}L)Li as a yellow powder (13.6 g, 100%). ^{1}H NMR (500 MHz, C_6D_6): δ ppm 6.58 (d, *J* = 4.0 Hz, 2H), 6.44 (d, *J* = 4.0 Hz, 2H), 1.96–1.89 (m, 6H), 1.87–1.75 (m, 12H), 1.74–1.58 (m, 12H); ^{13}C NMR (125 MHz, C_6D_6): δ ppm 174.88, 148.21 (d, *J* = 118.8 Hz), 143.78 (d, *J* = 121.2 Hz), 141.25, 140.10 (d, *J* = 118.8 Hz), 133.57, 131.33, 119.03, 116.78, 45.18, 39.76, 39.31, 31.68; ^{19}F NMR (470 MHz, THF): ppm −141.18 (dd, *J* = 23.0, 7.7 Hz, 2F), −155.33 (t, *J* = 23.0 Hz, 1F), −162.98 (td, *J* = 23.0, 7.7 Hz, 2F).

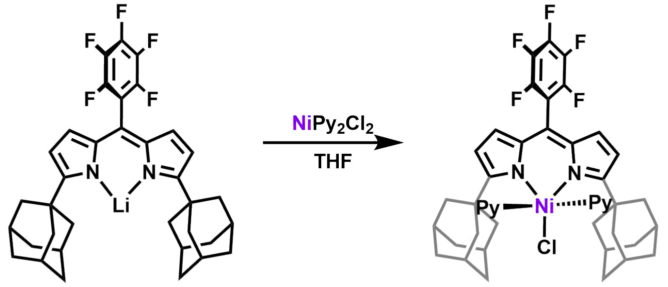

(^{AdF}L)NiCl(py)$_2$ (1): A suspension of $Ni(py)_2Cl_2$ (1.00 g, 3.47 mmol, 1.02 equiv) in 5 mL of THF was frozen. A solution of (^{AdF}L)Li (2.00 g, 3.42 mmol, 1.00 equiv) in 14 mL of THF was added to the frozen suspension. The mixture was warmed to room temperature and stirred overnight, during which time the solution turned from dark yellow to dark red. After 12 h, the volatiles were removed in vacuo. The resulting red solid was dissolved in benzene, frozen, and lyophilized. The resulting solid was washed with 20 mL of hexanes, dissolved in 20 mL of benzene, filtered through a Celite plug (20 cm^3), and lyophilized again to afford **(^{AdF}L)Ni(py)$_2$Cl (1)** as a dark red microcrystalline powder (2.21 g, 78%). Plate-shaped crystals suitable for X-ray diffraction were grown from a concentrated hexanes solution at −35 °C. ^{1}H NMR (500 MHz, C_6D_6): δ ppm 15.76 (s), 7.12 (s), 6.77 (s), 4.97 (s), 3.19 (s), 2.50 (d, *J* = 11.8 Hz), 2.39 (s), 1.88 (d, *J* = 11.8 Hz), −2.71 (s); ^{19}F NMR (470 MHz, C_6D_6): δ ppm −140.62 (d, *J* = 23.8 Hz), −141.01 (d, *J* = 23.9 Hz), −153.92 (t, *J* = 21.5 Hz), −162.37 (br. s). Anal. Calc. for $C_{44}H_{41}ClF_5N_4Ni$−Py: C 63.98, H 5.24, N 5.60; Found C 64.92, H 5.52, N 5.50. This compound slowly loses pyridine upon prolonged exposure to vacuum. The C content is high potentially due to residual pyridine.

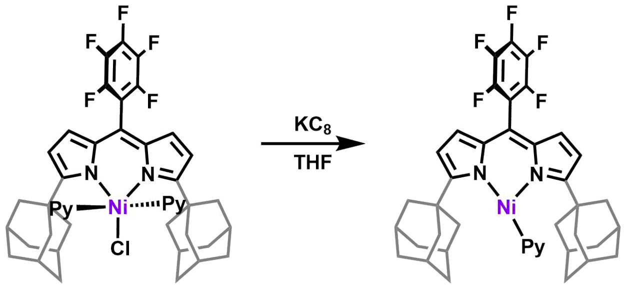

(^{AdF}L)Ni(py) (2): A solution of (^{AdF}L)Ni(py)$_2$Cl (**1**) (1.00 g, 1.21 mmol, 1.00 equiv) in 5 mL of THF was frozen. Potassium graphite (0.180 g, 1.33 mmol, 1.10 equiv) was added in one portion as soon as the solution started stirring, causing an instantaneous color change from deep red to brown. The solution was stirred for 3 min before filtering through a Celite plug (20 cm^3). The volatiles were then removed in vacuo. The resulting brown solid residue was dissolved in benzene, frozen, and lyophilized. The resulting solid was washed with 2 mL of hexanes, dissolved in 20 ml of benzene, filtered through a Celite plug (20 cm^3), and lyophilized again to afford **(^{AdF}L)Ni(py) (2)** as a brown microcrystalline powder (0.750 g, 87%). Needle-shaped X-ray quality crystals were grown by storing a concentrated hexanes solution at −35°C overnight. ^{1}H NMR (500 MHz, C_6D_6): δ ppm 45.10 (br. s), 38.57 (s), 20.41 (s), 12.63 (s), 4.06 (s), 1.30 (s); ^{19}F NMR (470 MHz, C_6D_6): δ ppm −138.67, −154.55, −162.25. EPR (toluene, 77 K): $S = ½$, $g_1 = 2.438$, $g_2 = 2.121$, $g_3 = 2.064$ (Figure 3). Anal. Calc. for $C_{40}H_{39}F_5N_3Ni$: C 67.15, H 5.49, N 5.87; Found C 67.42, H 5.54, N 5.90.

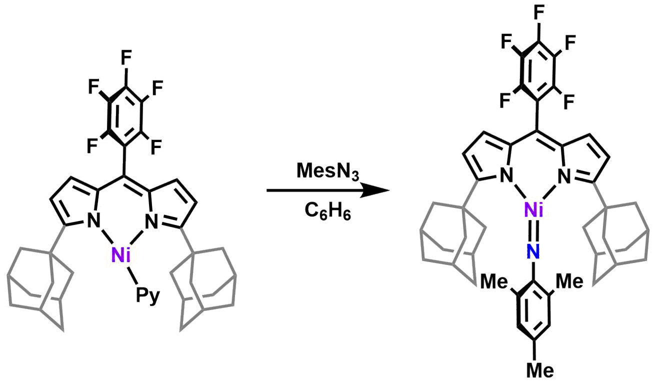

(^{AdF}L)Ni(NMes) (3): A solution of (^{AdF}L)Ni(py) (**2**) (100 mg, 0.140 mmol, 1.00 equiv) in 5 mL of benzene was frozen before another solution of $MesN_3$ (22.6 mg, 0.140 mmol, 1.00 equiv) in 3 mL of benzene was added dropwise. The mixture was warmed up to room temperature and stirred for 3 min, during which the solution changed from brown to deep pink along with vigorous effervescence. The solution was then lyophilized, and the solid residue was dissolved in 5 mL of hexanes and stored at −35°C overnight to give **(^{AdF}L)Ni(NMes) (3)** (34.5 mg, 32%) as dark pink

rod-shaped crystals suitable for X-ray diffraction. ^1H NMR (500 MHz, C_6D_6): this compound is ^1H NMR silent; ^{19}F NMR (470 MHz, C_6D_6): δ ppm −142.84, −152.11, −162.74. EPR (toluene, 77 K): $S_1 = ½$, $g_{11} = 2.238$, $g_{12} = 2.106$, $g_{13} = 1.940$; $S_2 = ½$, $g_{21} = 2.302$, $g_{22} = 2.128$, $g_{23} = 1.962$ (Figure 5a). Anal. Calc. for $C_{44}H_{45}F_5N_3Ni$: C 68.67, H 5.89, N 5.46; Found C 68.70, H 5.70, N 5.71.

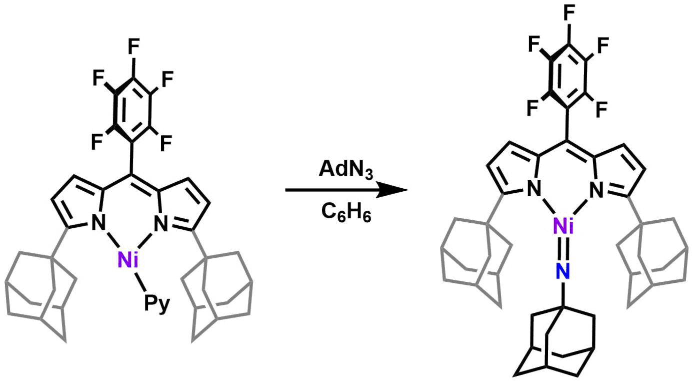

(^{AdF}L)Ni(NAd) (4): A solution of (^{AdF}L)Ni(py) (**3**) (100 mg, 0.140 mmol, 1.00 equiv) in 5 mL of benzene was frozen before another solution of AdN_3 (24.8 mg, 0.140 mmol, 1.00 equiv) in 3 mL of benzene was added dropwise. The mixture was warmed up to room temperature and stirred for 3 min, during which the solution changed from brown to deep pink along with vigorous effervescence. The solution was then lyophilized, and the solid residue was dissolved in 5 mL of hexanes and stored at −35°C overnight to give **(^{AdF}L)Ni(NAd) (4)** (42.9 mg, 39%) as dark pink rod-shaped crystals suitable for X-ray diffraction. ^1H NMR (500 MHz, C_6D_6): this compound is ^1H NMR silent; ^{19}F NMR (470 MHz, C_6D_6) δ ppm −140.75, −142.40, −153.73, −162.28, −162.72. EPR (toluene, 77 K): $S = ½$, $g_1 = 2.185$, $g_2 = 2.063$, $g_3 = 1.924$, $A(^{14}N)$= 21.3 G. (Figure 5b). Anal. Calc. for $C_{45}H_{49}F_5N_3Ni$: C 68.80, H 6.29, N 5.35; Found C 68.94, H 6.24, N 5.43.

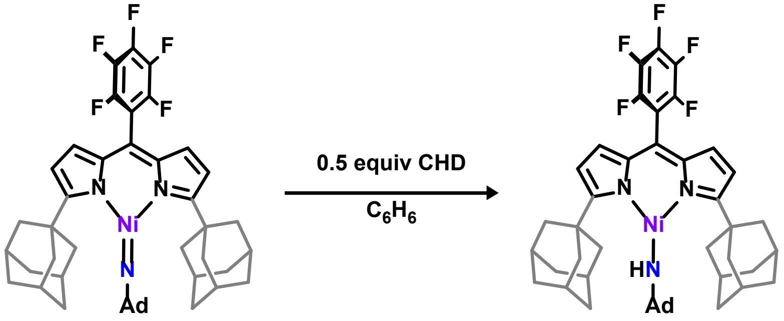

(^{AdF}L)Ni(NHAd) (5): A solution of (^{AdF}L)Ni(NAd) (**4**) (30.0 mg, 0.0382 mmol, 1.00 equiv) in 5 mL of benzene was frozen before another solution of 1,4-cyclohexadiene (18.0 μL, 0.190 mmol,

5.00 equiv) in 3 mL of benzene was added. The mixture was warmed up to room temperature and stirred for 3 min, during which the solution changed from deep to light pink. The solution was then lyophilized, and the solid residue was dissolved in 5 mL of hexanes and stored at −35°C overnight to give **(^{AdF}L)Ni(NHAd) (5)** (19.2 mg, 64%) as dark pink needle-shaped crystals suitable for X-ray diffraction. ^{1}H NMR (500 MHz, C_6D_6): δ ppm 15.76 (s), 7.12 (s), 6.77 (s), 4.97 (s), 3.19(s), 2.50 (d, *J* = 11.9 Hz), 2.39 (s), 1.88 (d, *J* = 11.9 Hz), −2.71 (s); ^{19}F NMR (470 MHz, C_6D_6) δ ppm −140.73, −141.13, −153.94, −162.42. Anal. Calc. for $C_{45}H_{50}F_5N_3Ni$: C 68.71, H 6.41, N 5.34; Found C 68.46, H 6.42, N 5.31.

Experimental procedure for reaction between CHD and 3

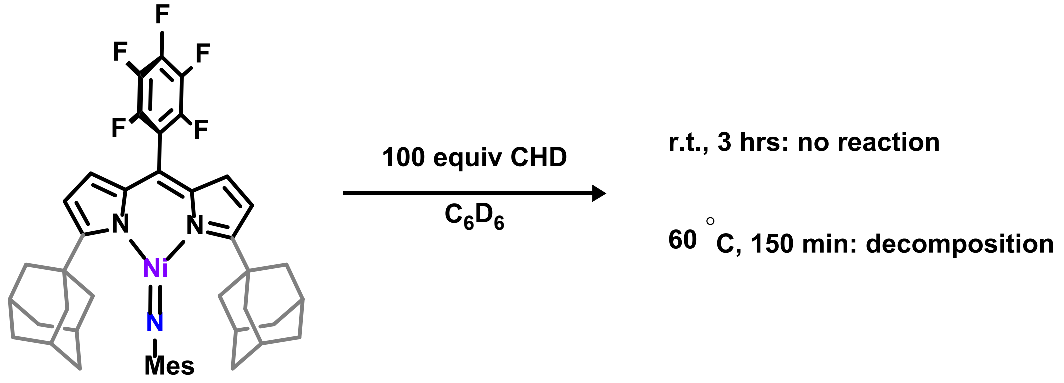

Compound **3** (20.0 mg, 0.026 mmol, 1 equiv) was dissolved in 0.4 ml of C_6D_6 in a J-Young tube in a glovebox filled with nitrogen atmosphere. To the solution, CHD (0.25 ml, 26.2 mmol, 100 equiv) was added dropwise. The J-Young tube was then sealed and let sit at room temperature for 3 hours before a ^{19}F NMR spectrum was taken. Then, the J-Young tube was heated to 60 °C in an oil bath for 150 min before the second ^{19}F NMR spectrum was taken to analyze the nickel containing species.

In a separate J-Young tube, **3** (20.0 mg, 0.026 mmol, 1 equiv) was dissolved in 0.65 ml of C_6D_6 in a J-Young tube and heated under nitrogen for 150 min before a ^{19}F NMR spectrum was taken as control experiment.

Experimental procedure for reaction between CHD and 4

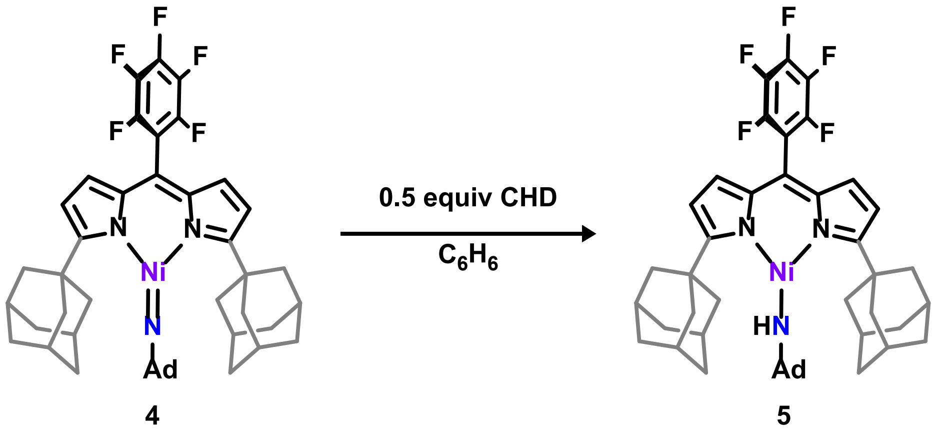

Compound **4** (20.0 mg, 0.025 mmol, 1 equiv) was dissolved in 0.4 ml of C_6D_6 in a J-Young tube in a glovebox filled with nitrogen atmosphere. A stock solution of CHD (12 µL, 0.127 mmol, 5 equiv) in 2.5 ml of C_6D_6 was made, and 0.25 ml of such stock solution was added to the J-Young

tube before it was sealed. Immediate color change from deep pink to light pink was observed and a ^{19}F NMR spectrum was taken to analyzed the nickel containing species.

Experimental procedure for reaction between toluene and 4

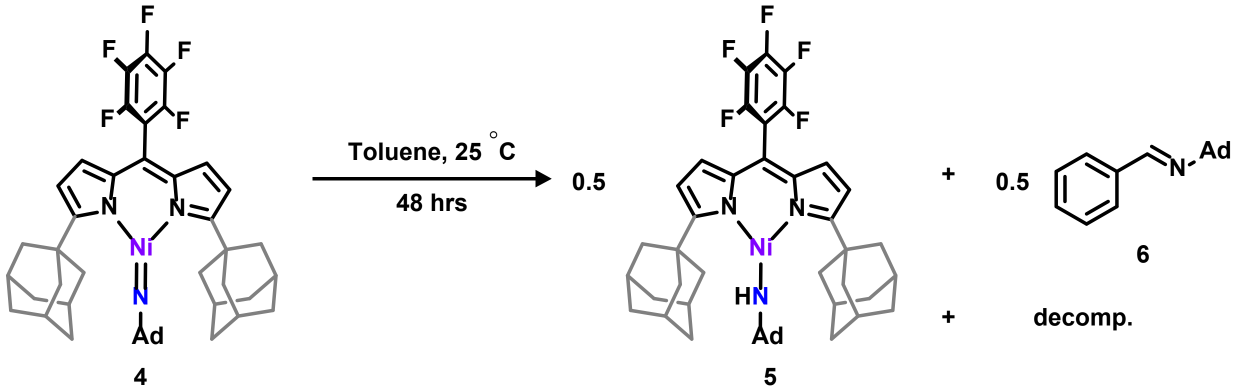

Compound **4** (20.0 mg, 0.025 mmol, 1 equiv) was dissolved in 0.6 ml of toluene in a J-Young tube in a glovebox filled with nitrogen atmosphere. To this solution trifluorotoluene (12 μL, 0.100 mmol, 4 equiv) was added using a microsyringe as an internal standard. The J-Young tube was sealed and ^{19}F NMR spectra were taken after 10 min, 3 hours, 24 hours and 48 hours, which showed the completion of the reaction after 2 days. The resulting mixture was passed through a silica plug (~5 cm^3) using 200 ml of diethyl ether to ensure complete elution of product and proteo ligand. After evaporation of the solvent, a ^1H NMR spectrum was taken in $CDCl_3$.

Experimental procedure for concentration profile of 4 during reaction between 4 and toluene

Compound **4** (40.0 mg, 0.050 mmol, 1 equiv) was dissolved in 0.6 ml of toluene in a J-Young tube in a glovebox filled with nitrogen atmosphere. To this solution trifluorotoluene (24 μL, 0.200 mmol, 4 equiv) was added using a microsyringe as an internal standard.The J-Young tube was sealed and ^{19}F NMR spectra were taken during the course of the reaction at various time points.

Experimental procedure for reaction between CHD and 3

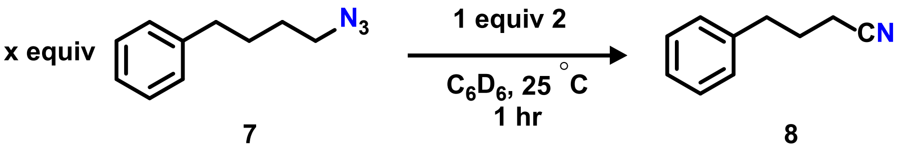

Compound **2** (20.0 mg, 0.028 mmol, 1 equiv) was dissolved in 0.2 ml of C_6D_6 in a vial with a magnetic stir bar. This solution was stirred, and **7** (5.0 mg, 0.028 mmol, 1 equiv) in 0.4 ml of C_6D_6

was slowly added dropwise. The reaction mixture immediately changed color to deep pink while vigorous effervescence was observed. After 5 mins, the effervescence ceased, and the solution color reverted to deep brown. The solution was transferred to a J-Young tube and NMR spectra (^{19}F and ^{1}H) were acquired. The same procedure was repeated with 3, 5, and 6 equiv of **7**.

Experimental procedure for catalytic reaction between 7 and 20 mol% of 2

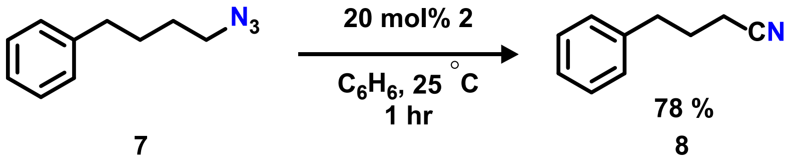

Compound **2** (16.3 mg, 0.023 mmol, 1 equiv) was dissolved in 0.2 ml of C_6H_6 in a vial with a magnetic stir bar. This solution was stirred, and **7** (20.0 mg, 0.114 mmol, 5 equiv) in 0.4 ml of C_6H_6 was slowly added dropwise. The reaction mixture immediately changed color to deep pink while vigorous effervescence was observed. After 20 mins, the effervescence ceased, and the solution color reverted to deep brown. Product **8** (13.0 mg, 78%) was separated using preparatory thin layer chromatography. Spectral data were consistent with previously reported characterization of the product.[5]

^{1}H NMR (500 MHz, $CDCl_3$) δ/ppm: 7.41–7.34 (m, 2H), 7.32–7.27 (m, 1H), 7.27–7.22 (m, 2H), 2.85–2.77 (m, 2H), 2.32 (t, J = 7.2 Hz, 2H), 2.08–1.92 (m, 2H); ^{13}C{^{1}H} NMR (126 MHz, $CDCl_3$) δ/ppm: 139.60, 128.39, 128.21, 126.21, 119.34, 34.10, 26.66, 16.06.

Figure S1. Frozen solution EPR spectrum of (^{AdF}L)Ni(py) (2)

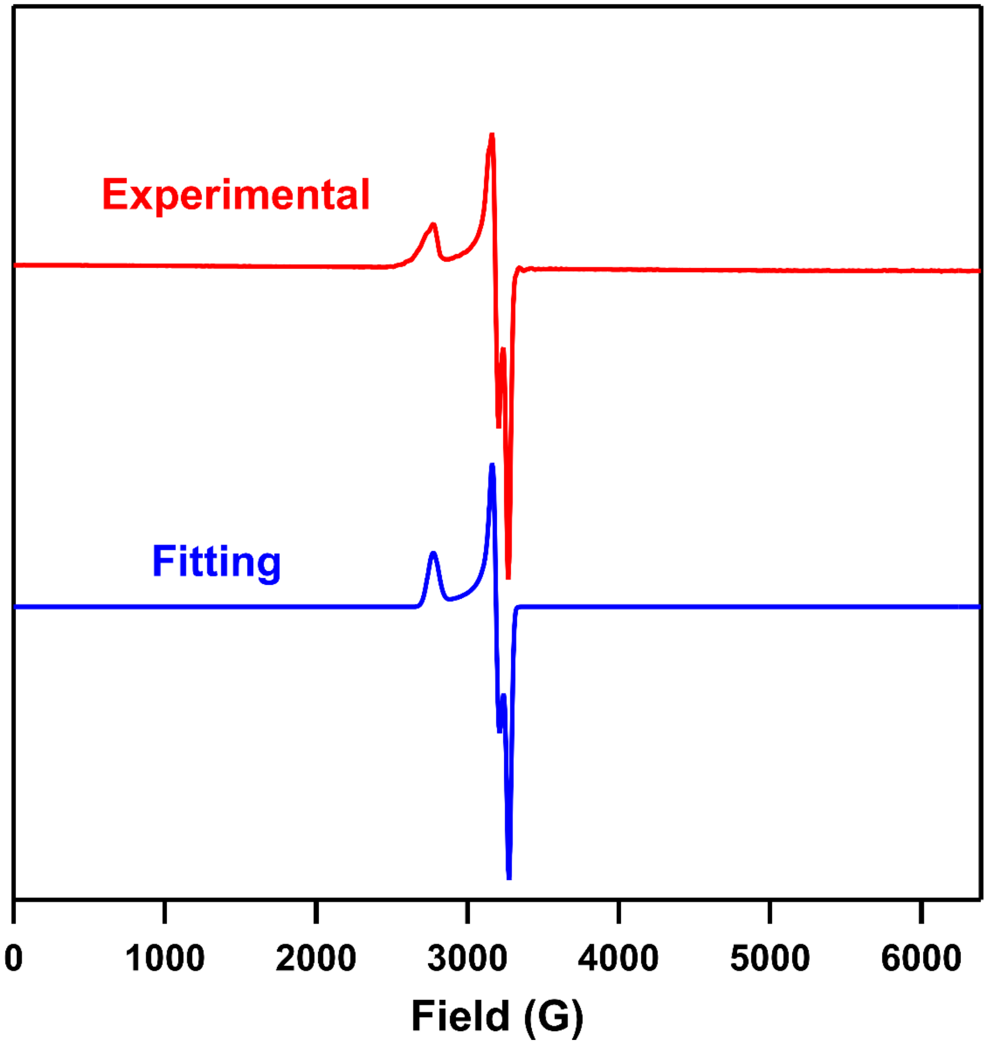

Figure S1. Frozen solution EPR spectrum of (^{AdF}L)Ni(py) (**2**) collected at 77 K in toluene (Red). Blue line represents a fit of the data using the program EasySpin.[3] Fitting parameters: $S = ½$, $g_1 = 2.438$, $g_2 = 2.121$, $g_3 = 2.064$.

Figure S2. Frozen solution EPR spectrum of (^{AdF}L)Ni(NMes) (3)

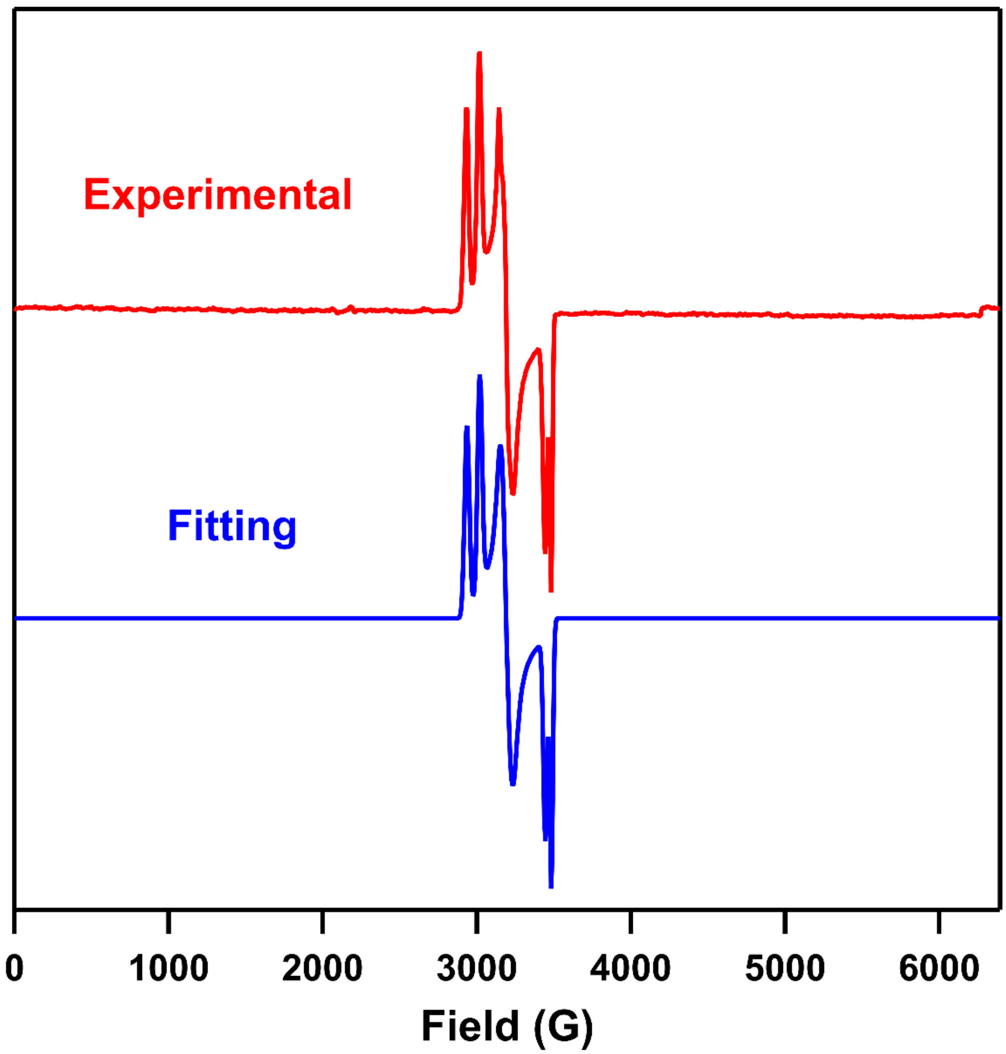

Figure S2. Frozen solution EPR spectrum of (^{AdF}L)Ni(NMes) (**3**) collected at 77 K in toluene (Red). Blue line represents a fit of the data using the program EasySpin.[3] Fitting parameters: S_1 = ½ , g_{11} = 2.238, g_{12} = 2.106, g_{13} = 1.940; S_2 = ½ , g_{21} = 2.302, g_{22} = 2.128, g_{23} = 1.962.

Figure S3. Frozen solution EPR spectrum of (^{AdF}L)Ni(NAd) (4)

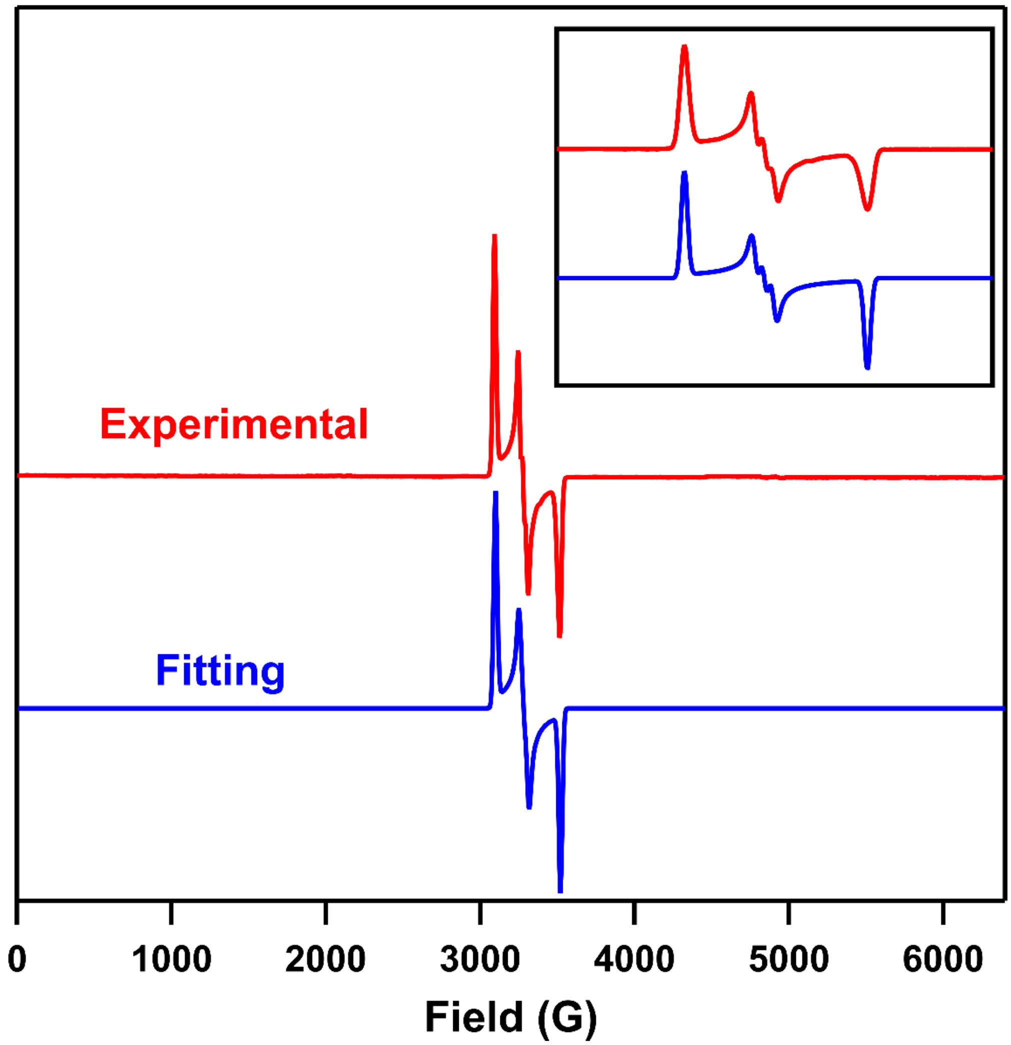

Figure S3. Frozen solution EPR spectrum of (^{AdF}L)Ni(NAd) (**4**) collected at 77 K in toluene (Red). Blue line represents a simulation of the data using the program EasySpin.[3] Fitting parameters: S_1 = ½ , g_1 = 2.238, g_2 = 2.106, g_3 = 1.940, $A(^{14}N)$= 21.3 G.

Figure S4. Magnetization data of (^{AdF}L)Ni(NMes) (3)

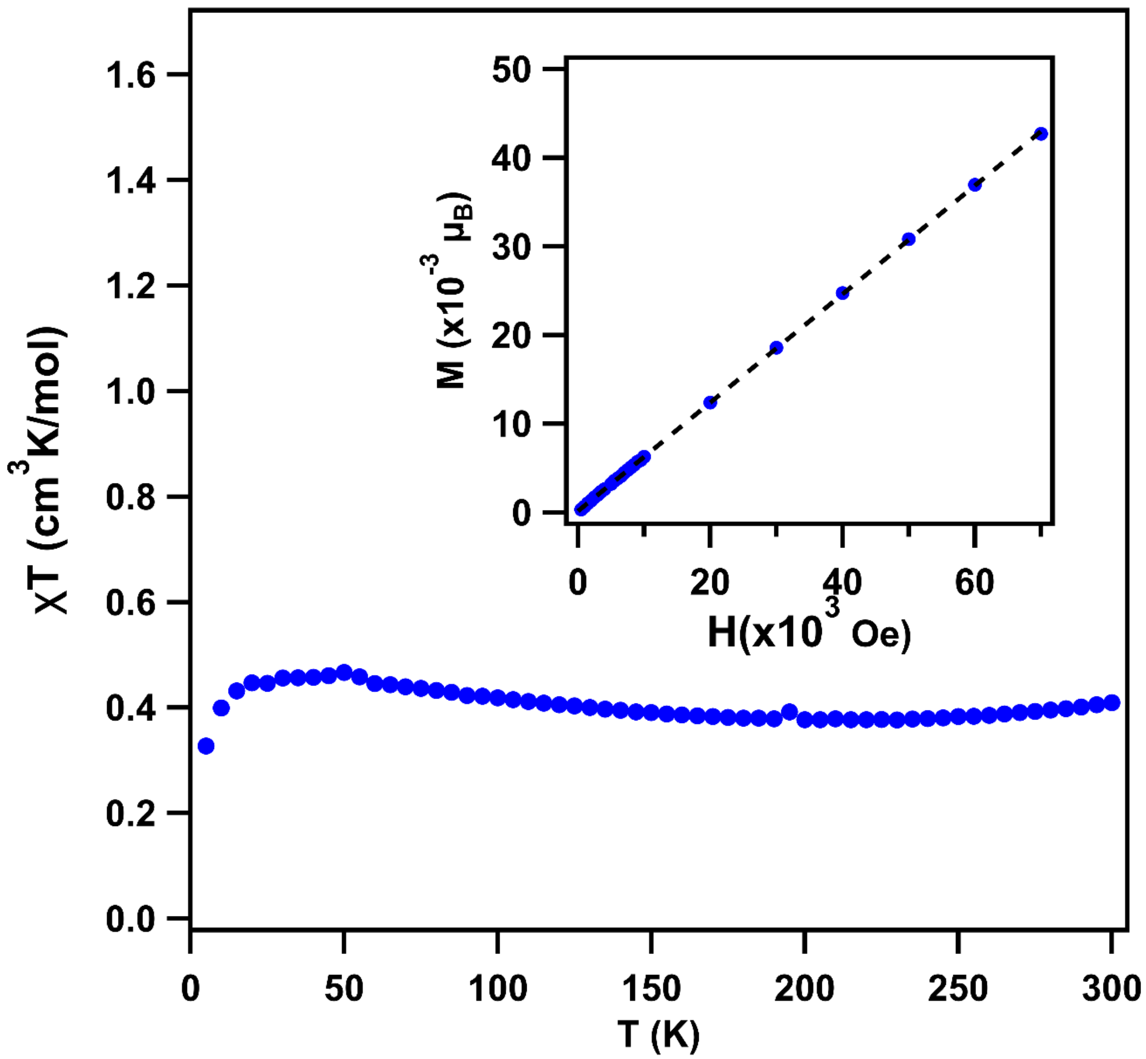

Figure S4. Variable temperature susceptibility data collected at 1.0 T. (inset) Magnetization versus field collected at 100 K – linear plot reflects the sample is free from ferromagnetic impurities.

Figure S5. Magnetization data of (^{AdF}L)Ni(NAd) (4)

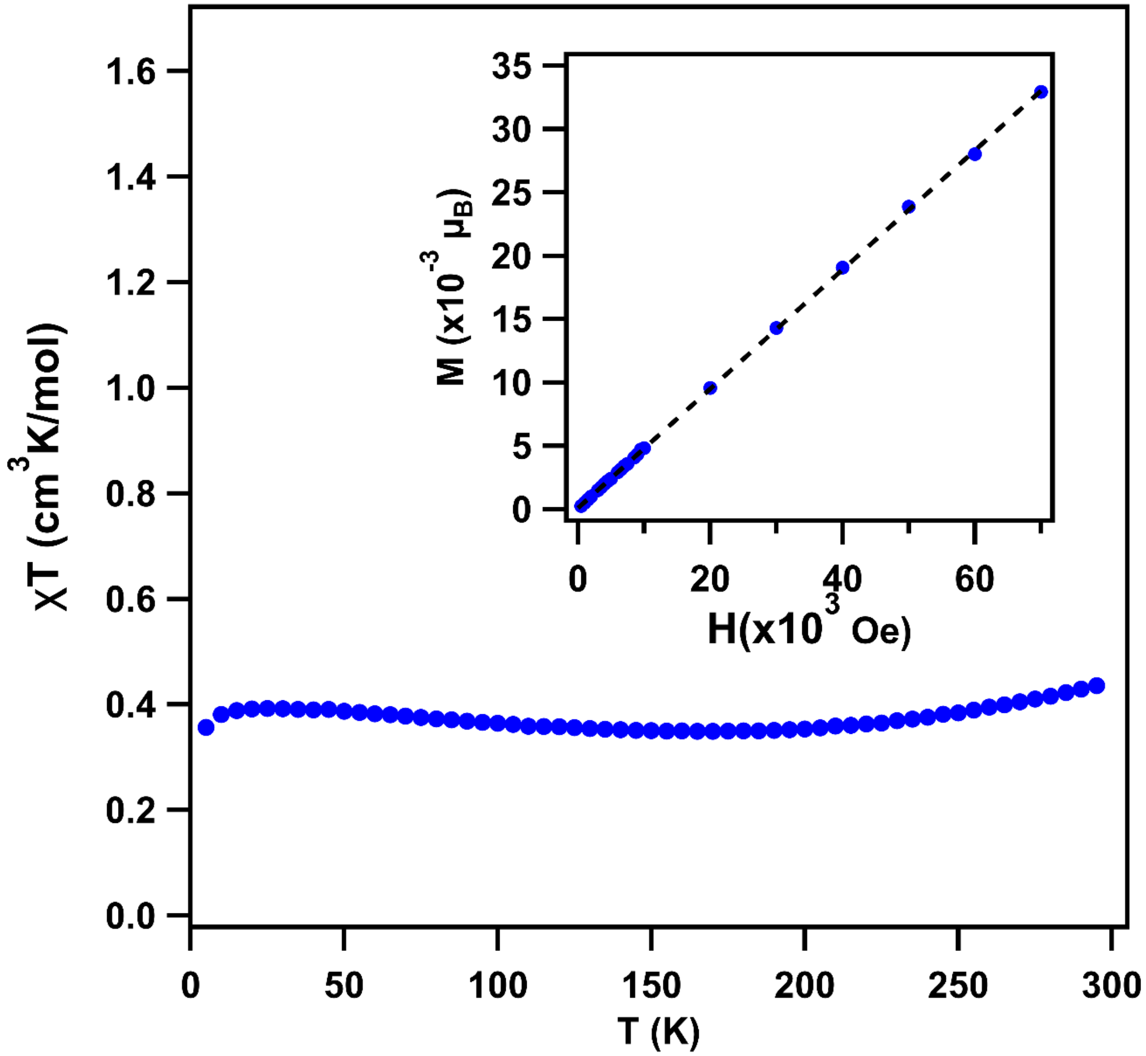

Figure S5. Variable temperature susceptibility data collected at 1.0 T. (inset) Magnetization versus field collected at 100 K – linear plot reflects the sample is free from ferromagnetic impurities.

Figure S6. Cyclic voltammogram of (^{AdF}L)Ni(NMes) (3)

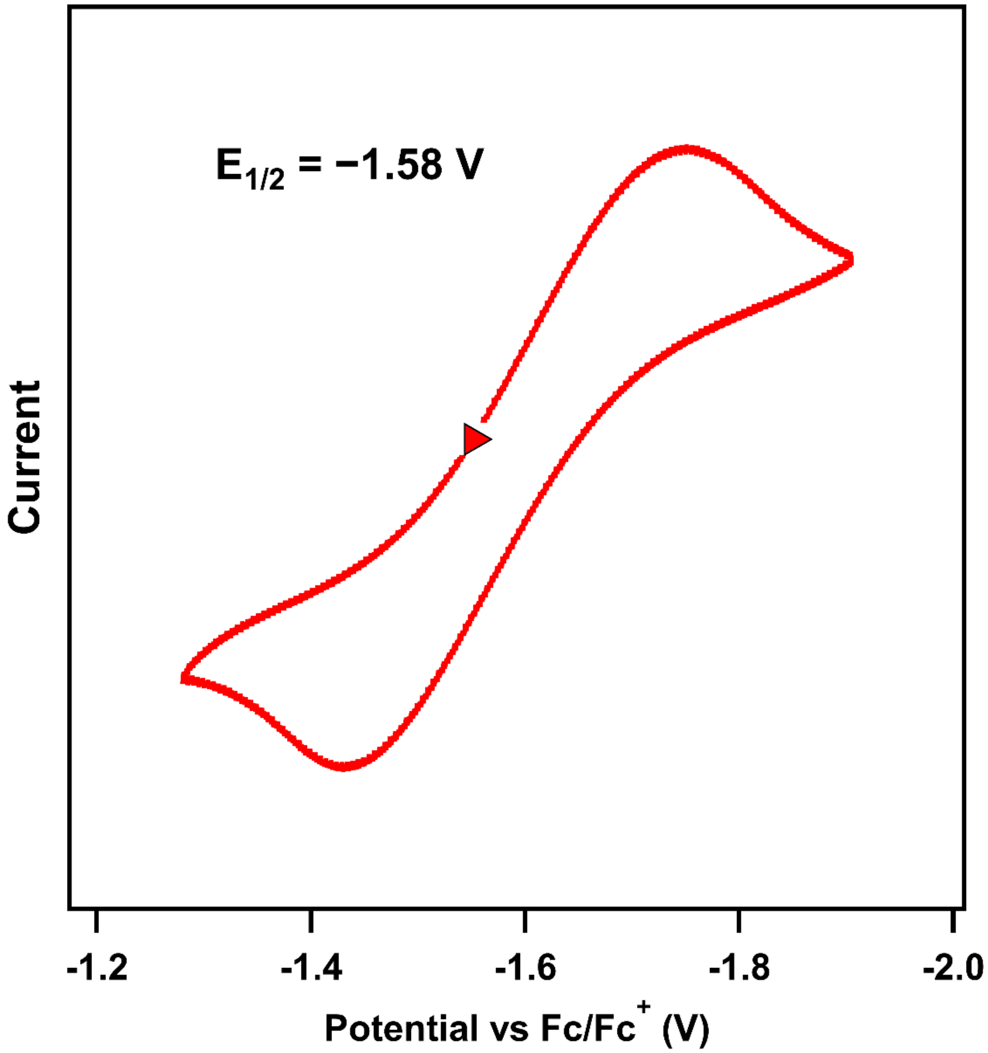

Figure S6. Cyclic voltammograms of **3** obtained in THF at 25 °C, with 0.1 M (^{n}Bu$_4$N)(PF$_6$); 100 mV/s; referenced to [Cp$_2$Fe]$^{+/0}$ couple; OCP = −1.25 V; ΔE = 310 mV.

Figure S7. Relevant bond metrics in all Ni Imides

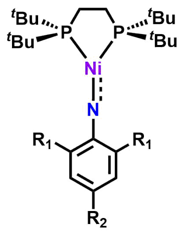

R_1 = Me, R_2 = Me
R_1 = ^{i}Pr, R_2 = H

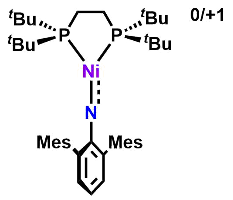

Mes:	DIPP:	0:	+1:
Ni-N 1.703(4)	Ni-N 1.702(2)	Ni-N 1.697(2)	Ni-N 1.674(3)
Ni-N-C 180	Ni-N-C 162.8(2)	Ni-N-C 168.98(12)	Ni-N-C 178.4(3)

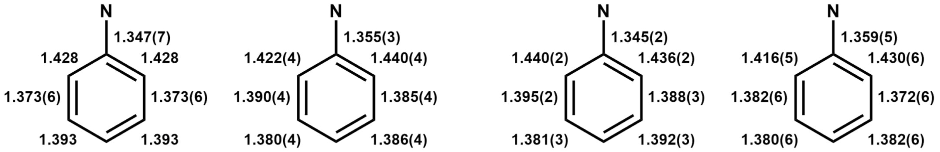

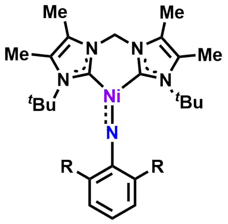

R = DIPP or Mes

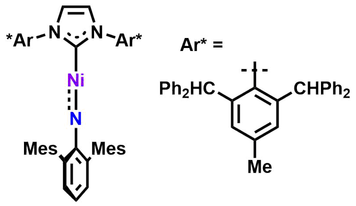

Mes:	DIPP:	Mes:
Ni-N 1.732(4)	Ni-N 1.718(2)	Ni-N 1.663(3)
Ni-N-C 127.3(3)	Ni-N-C 170.0(2)	Ni-N-C 171.6(3)

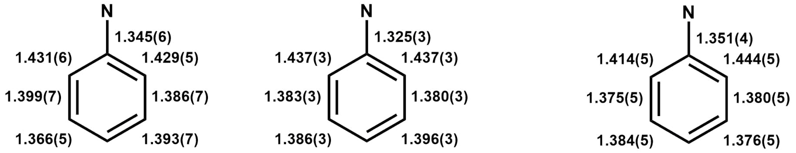

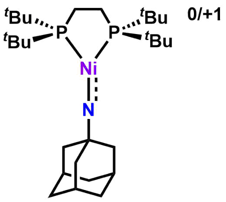

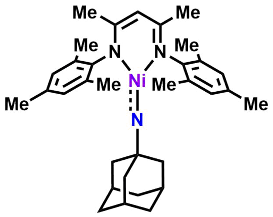

0:	+1:	
Ni-N 1.673(2)	Ni-N 1.657(5)	Ni-N 1.662(2)
Ni-N-C 163.0(2)	Ni-N-C 165.2(4)	Ni-N-C 164.5

Figure S8. ^{19}F NMR spectra of reaction between CHD and 3 at 25 °C

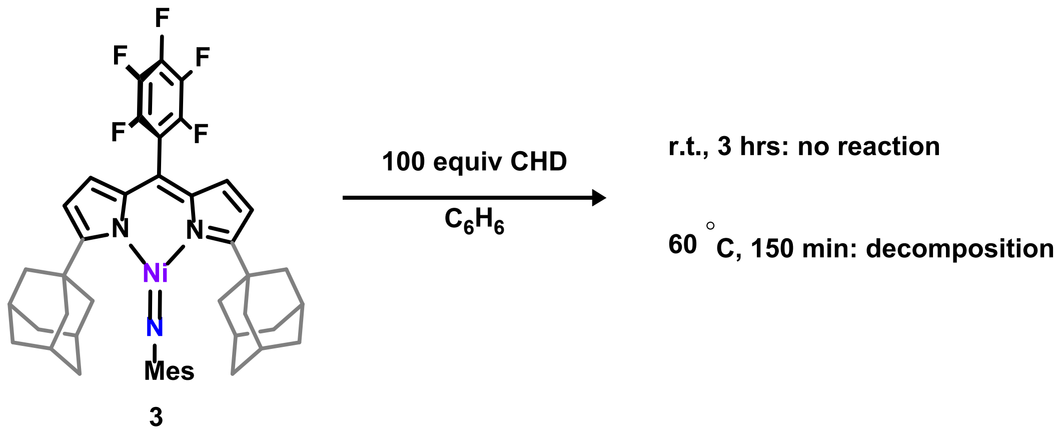

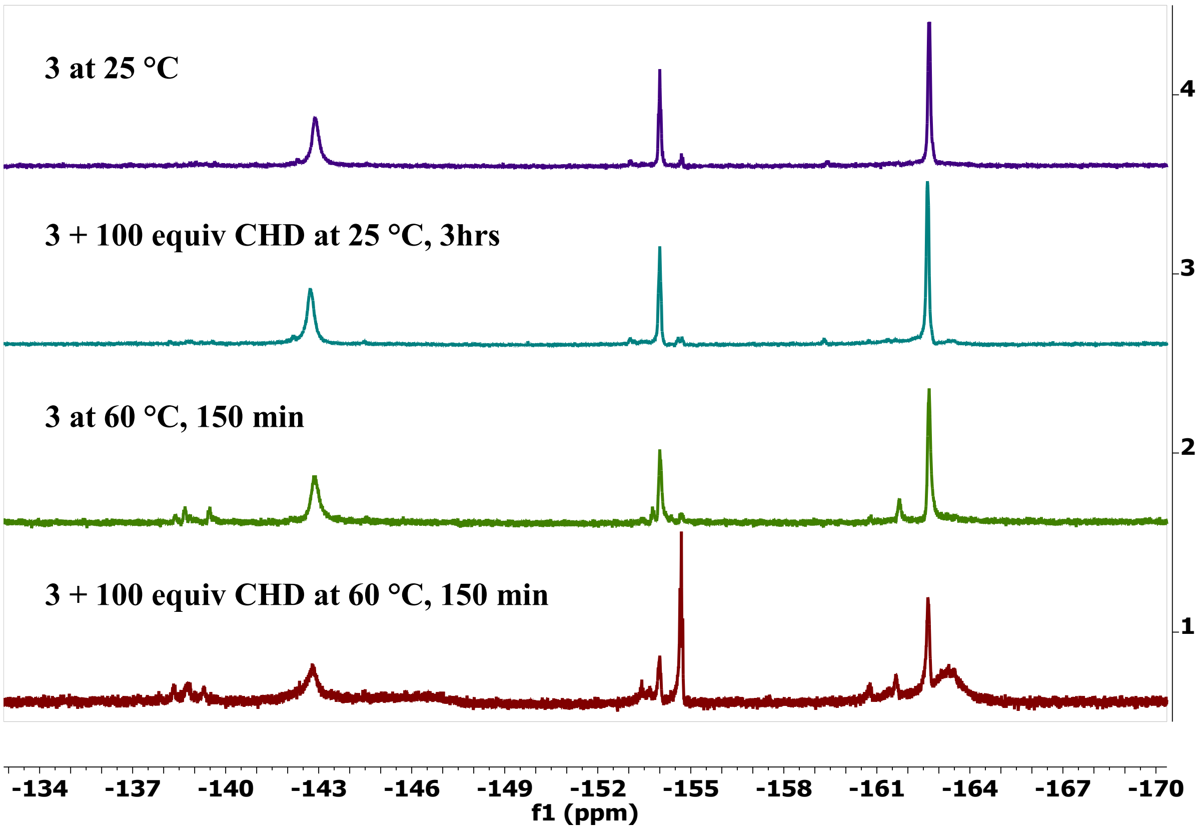

Figure S8. ^{19}F NMR spectra of reaction between CHD and 3, all peaks are referenced to $BF_3{\cdot}Et_2O$ (−153 ppm).

Figure S9. ^{19}F NMR spectra of reaction between CHD and 4 at 25 °C

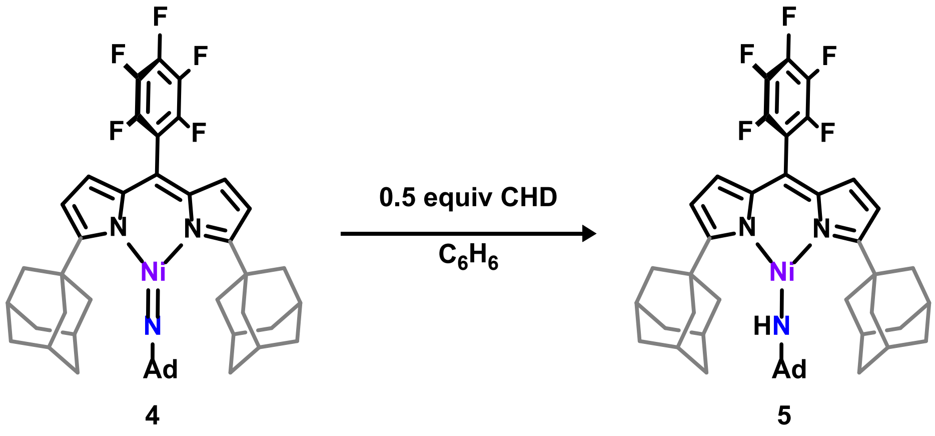

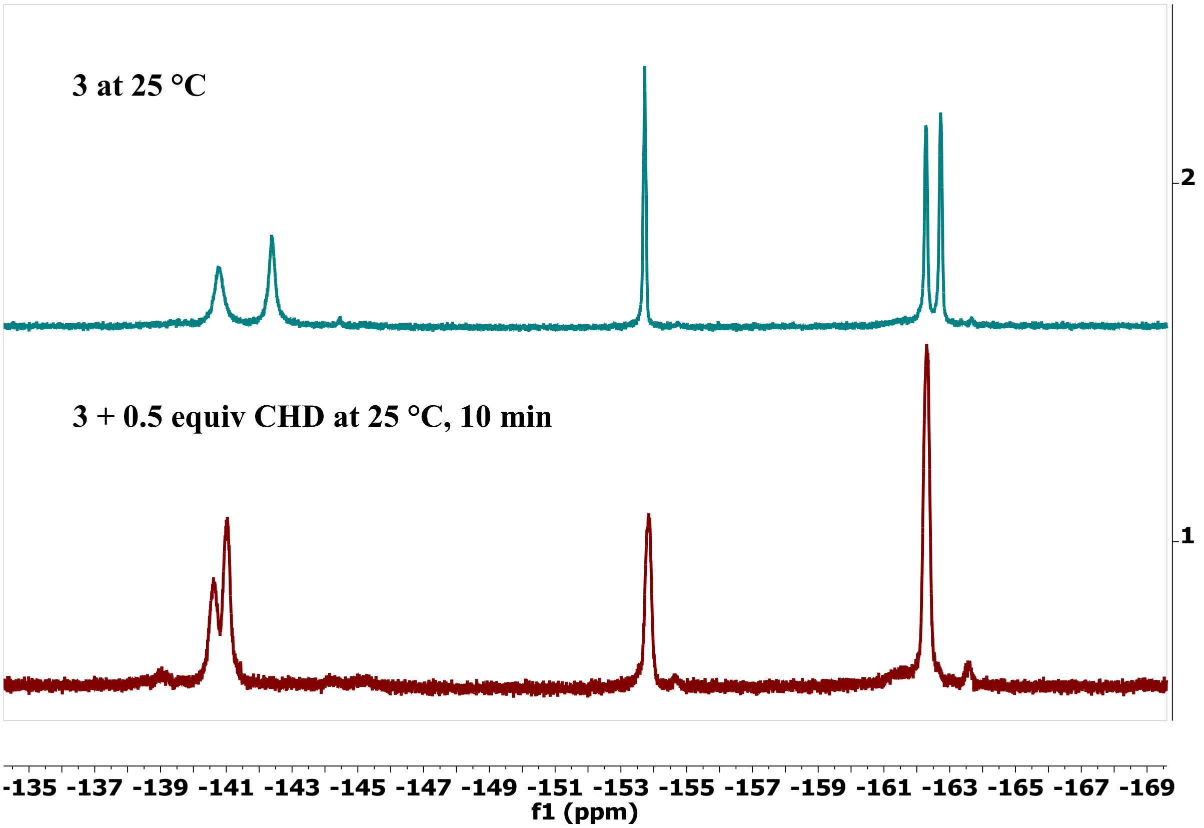

Figure S9. ^{19}F NMR spectra of reaction between CHD and **4**, all peaks are referenced to $BF_3{\cdot}Et_2O$ (−153 ppm).

Figure S10. ^{19}F NMR and ^{1}H NMR spectra of reactions between toluene and 4 at 25 °C

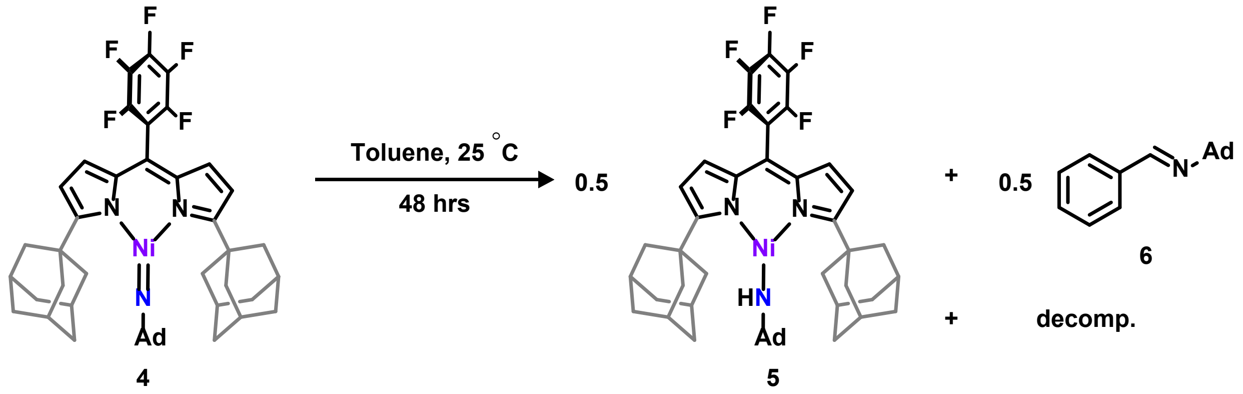

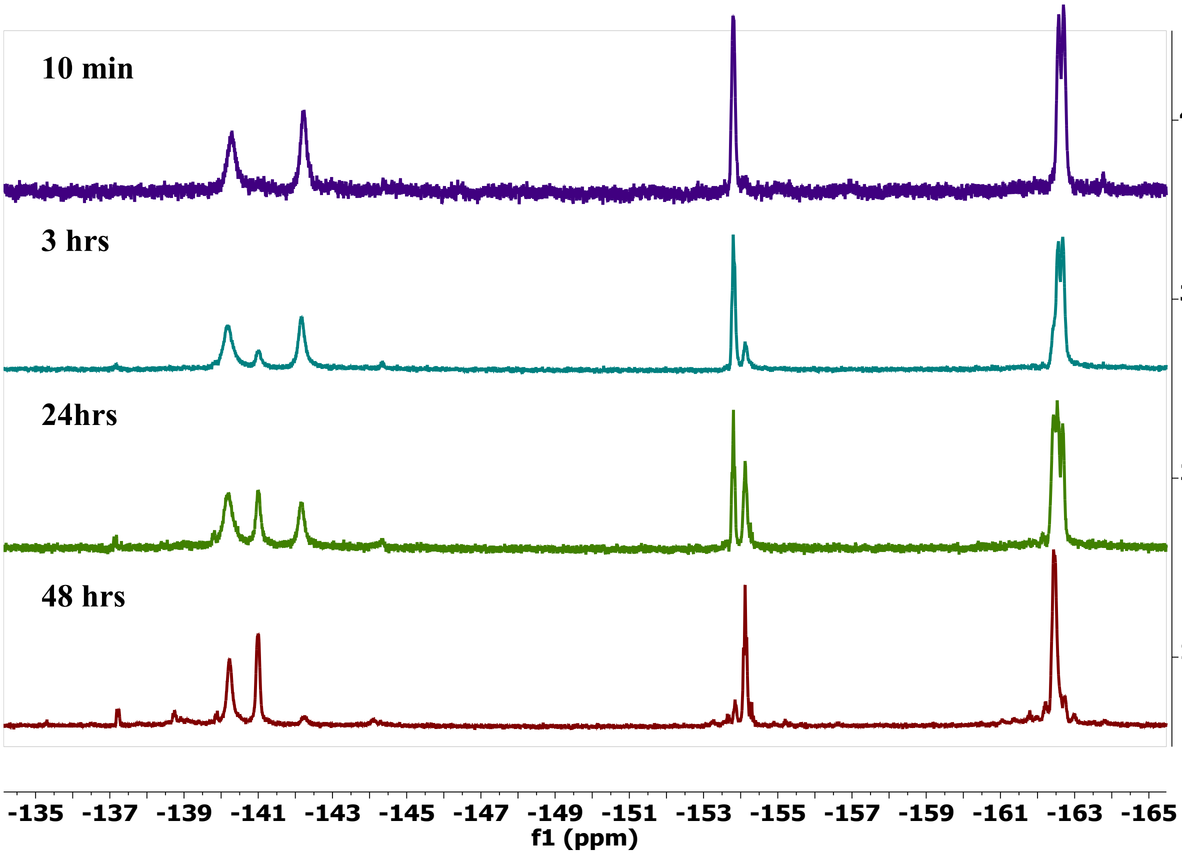

Figure S10-1. ^{19}F NMR spectra of reaction between toluene (solvent) and **4**, all peaks are referenced to $BF_3 \cdot Et_2O$ (−153 ppm). Concentration of **5** is determined to be half of the initial concentration of **4** using trifluorobenzene as internal standard.

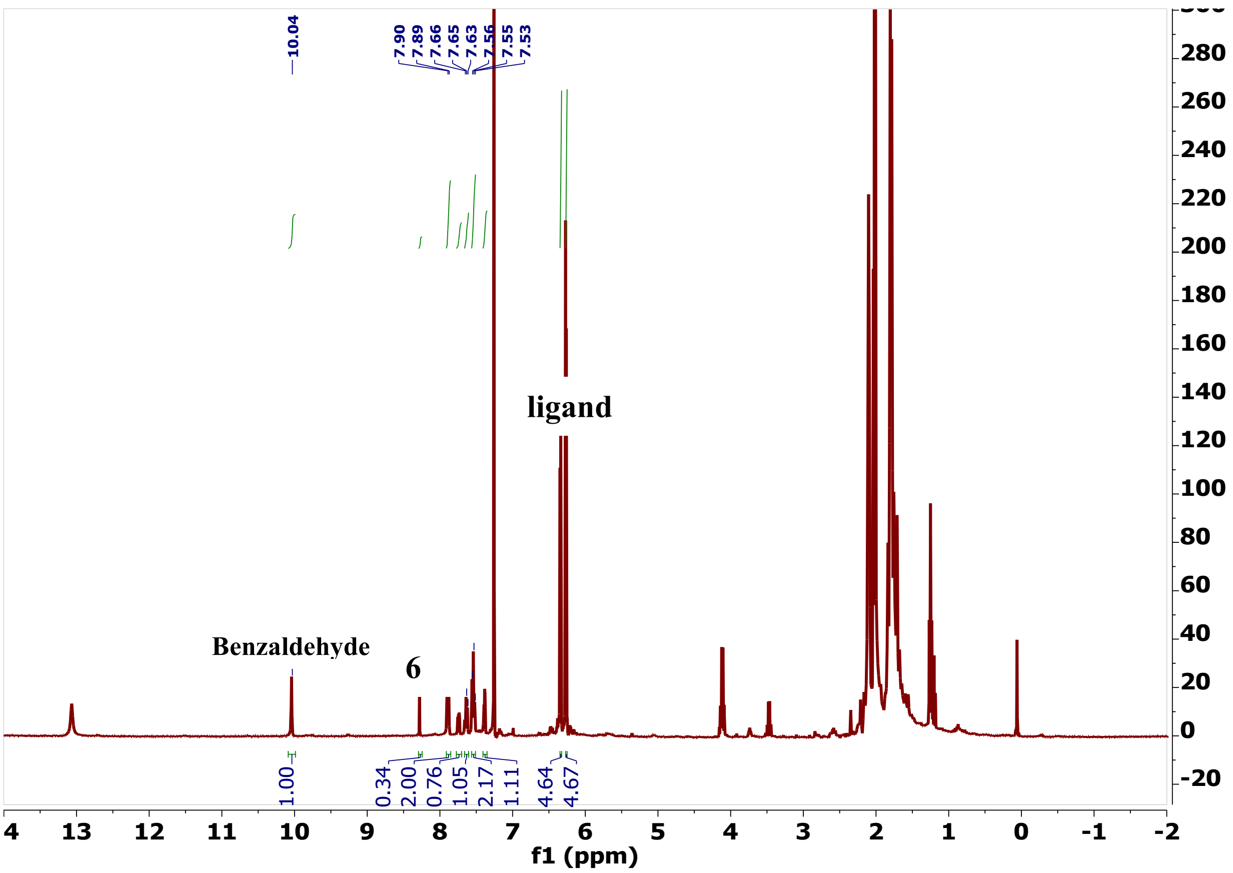

Figure S10-2. ^{1}H NMR spectra of quenched reaction between toluene (solvent) and **4** by passing through a silica plug using ample amount of Et_2O. The integration shows the ratio of 1:2 between imine product **6** (including hydrolysis into benzaldehyde) and free proteo ligand.[6]

Figure S11. Concentration profile of reaction between toluene and 4 by ^{19}F NMR spectroscopy at 25 °C

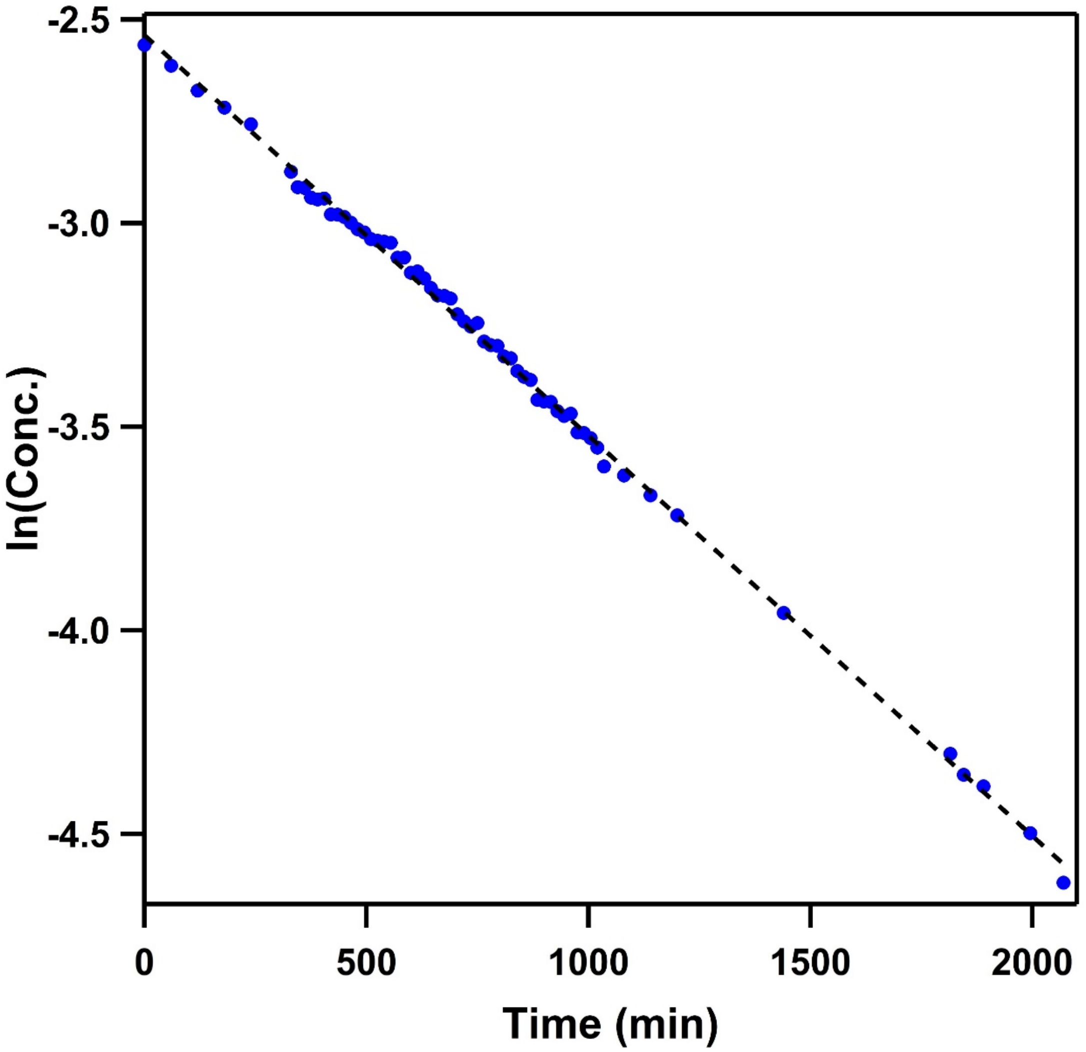

Figure S11. Concentration profile of **4** during reaction between toluene and **4** by ^{19}F NMR spectroscopy. Trifluorotoluene is used as internal standard. Linear relationship between ln([**4**]) and time shows that the reaction has pseudo first order dependence on the concentration of **4**.

Figure S12. ^{1}**H,** ^{13}**C, and** ^{19}**F NMR spectra of reaction between 7 and 2 at 25 °C**

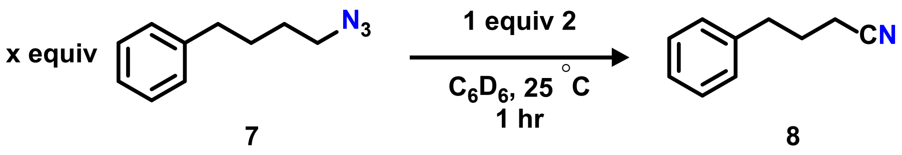

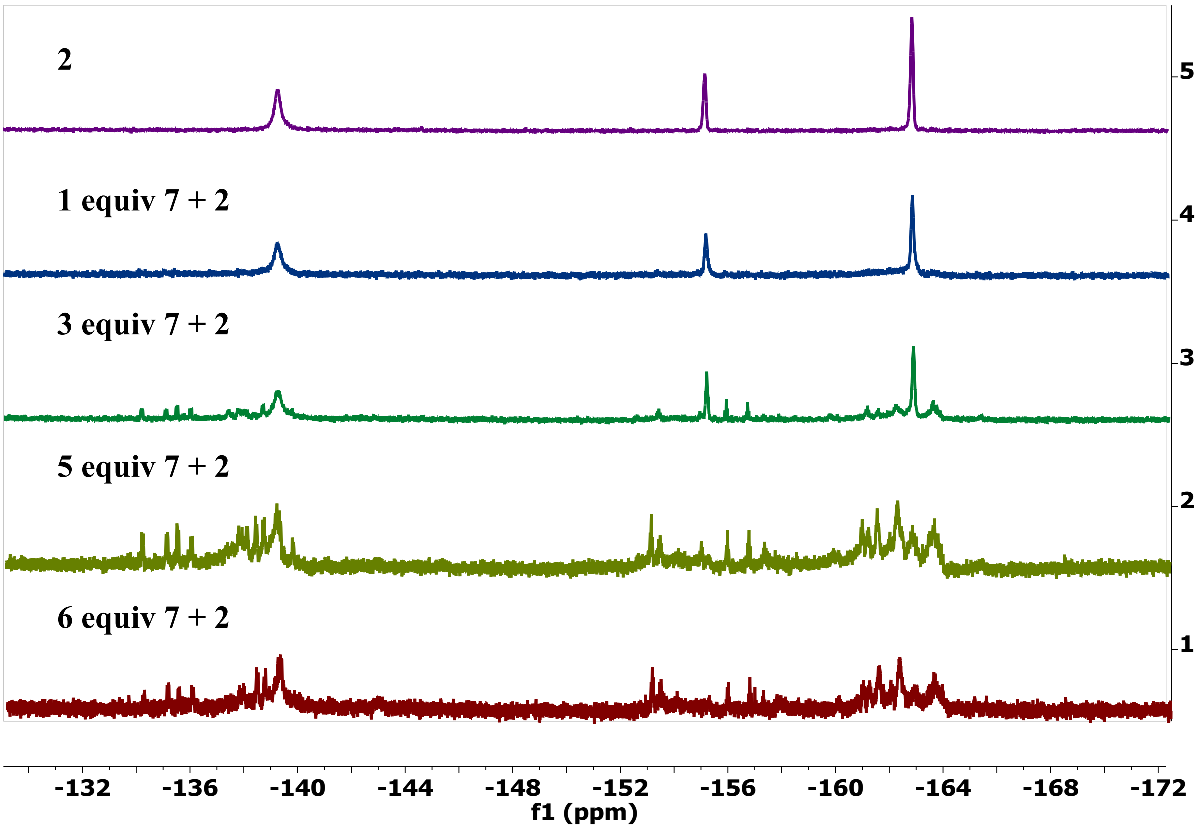

Figure S12-1. ^{19}F NMR spectra of reaction between multiple equivalence of **7** and **2**. The ^{19}F NMR stack shows complete regeneration of **2** with 1 equiv of **7**, partial regeneration of **2** with 3 equiv of **7**, and complete decomposition of **2** with 5 or 6 equiv of **7**. The reaction is carried out by slow addition of **7** to a C_6D_6 solution of **2**.

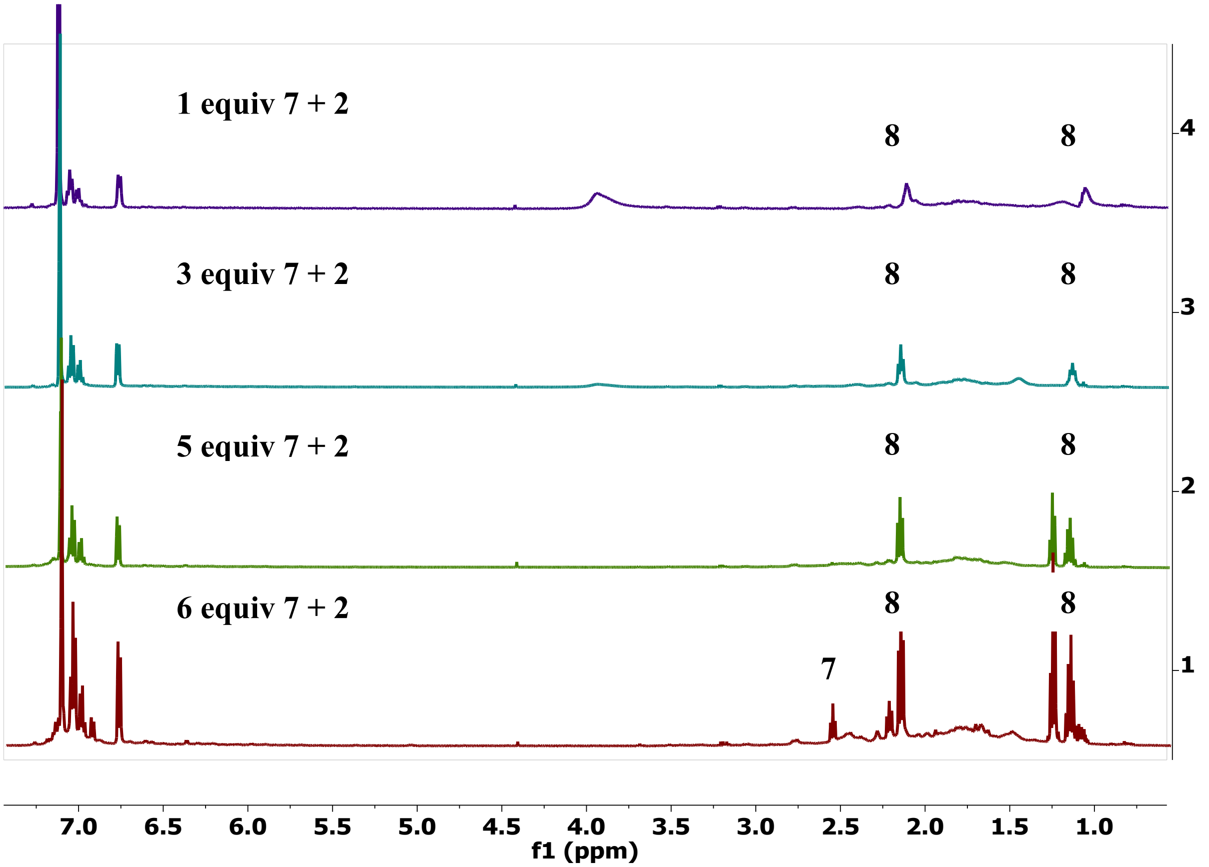

Figure S12-2. ^{1}H NMR spectra of reaction between multiple equivalence of **7** and **2**. The ^{1}H NMR stack shows complete consumption of starting material up to when 5 equiv of 7 was added. Note the peak correspond to 8 at around 1.2 ppm was not visible potentially (1 and 3 equiv of 7) due to low concentration and interaction with paramagnetic center.

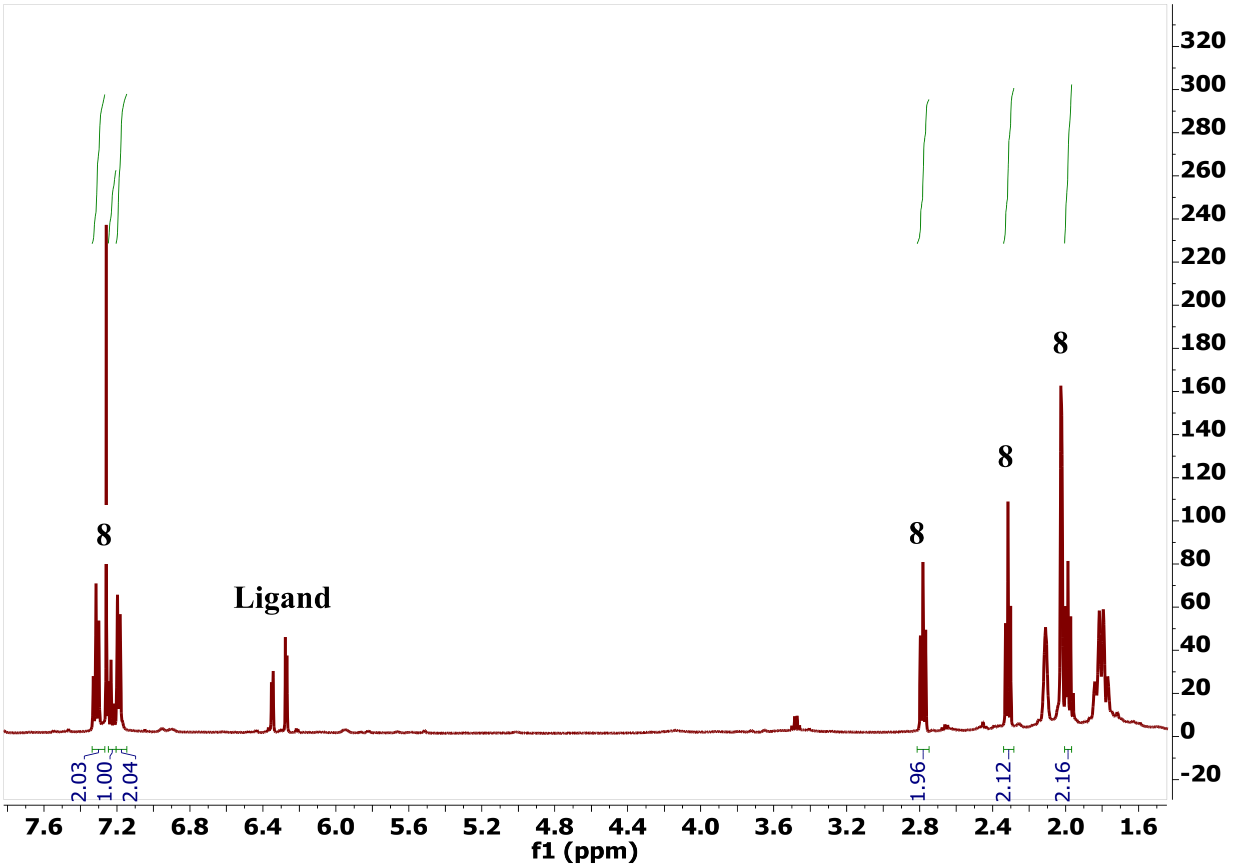

Figure S12-3. ^{1}H NMR spectra of quenched reaction between 1 equiv of **7** and **2** by passing reaction mixture through a silica plug using ample amount of Et_2O. The labeled signals show the generation of **8**.

Figure S12-4. ^{1}H NMR of phenylbutylnitrile (8)

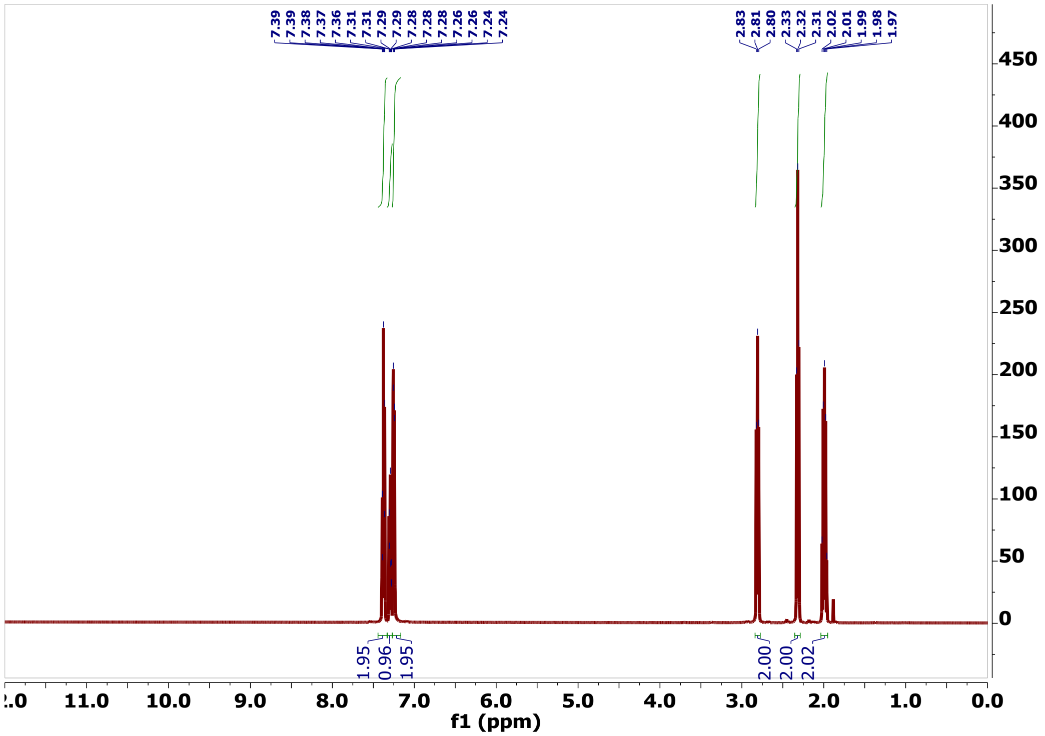

Figure S12-5. ^{13}C NMR of phenylbutylnitrile (8)

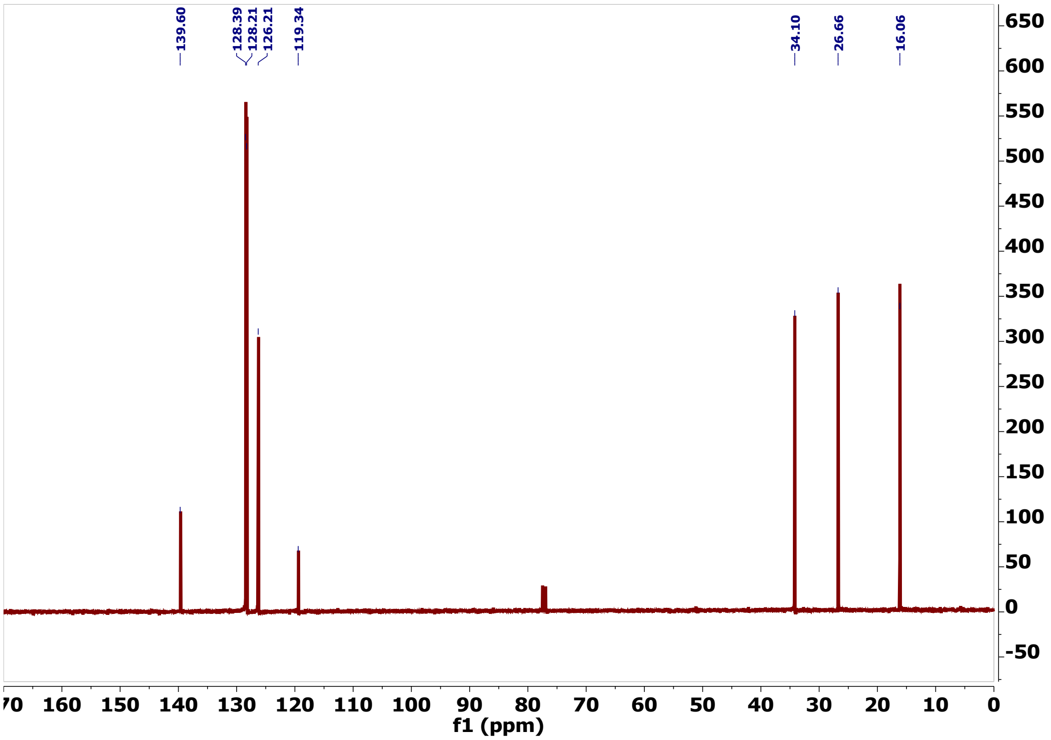

Figure S13. Progressive N K-edge XAS scans obtained for 4

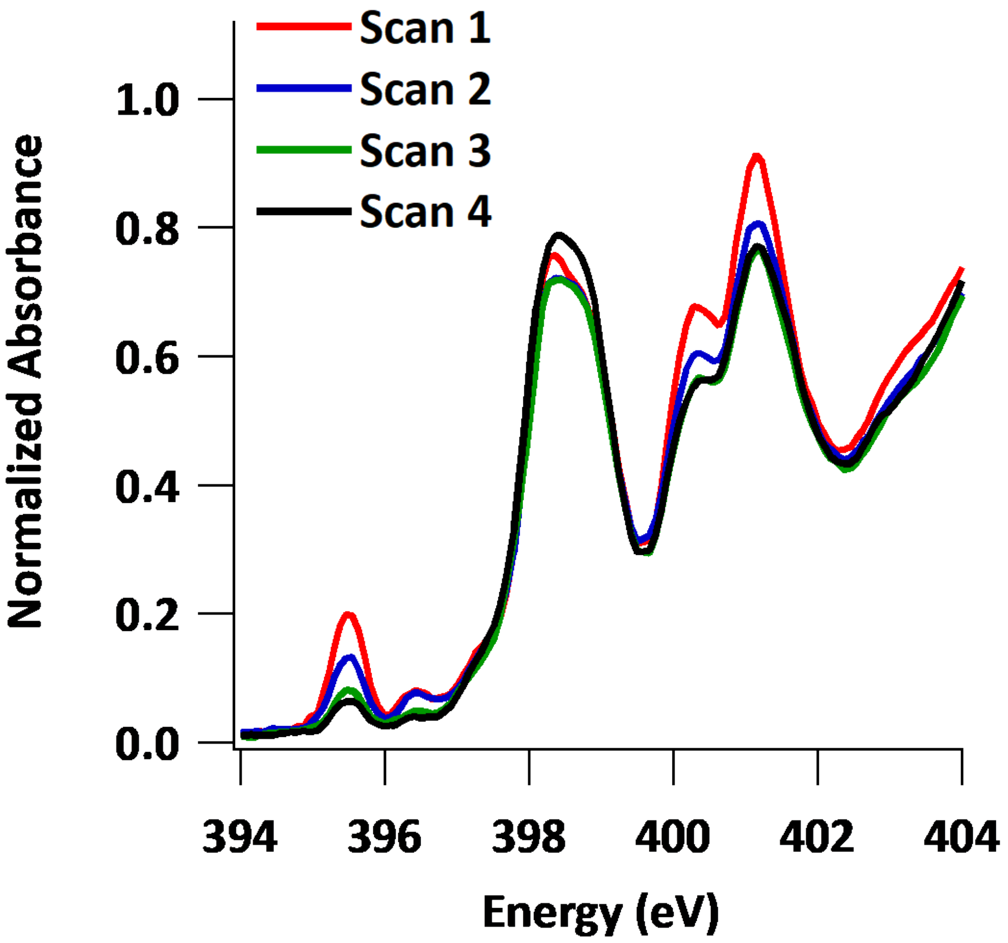

Figure S13. Progressive N K-edge XAS scans obtained for **4** at the same sample spot demonstrate that the pre-edge peaks assigned to the NAd nitrogen are highly susceptible to photodamage. Total duration of the measurement was 1400 seconds, with passage over the ca. 395.5 eV maximum occurring at 143, 493, 843, and 1193 seconds of total irradiation.

Figure S14. TD-DFT calculated N K-edge XAS for 3 and 4

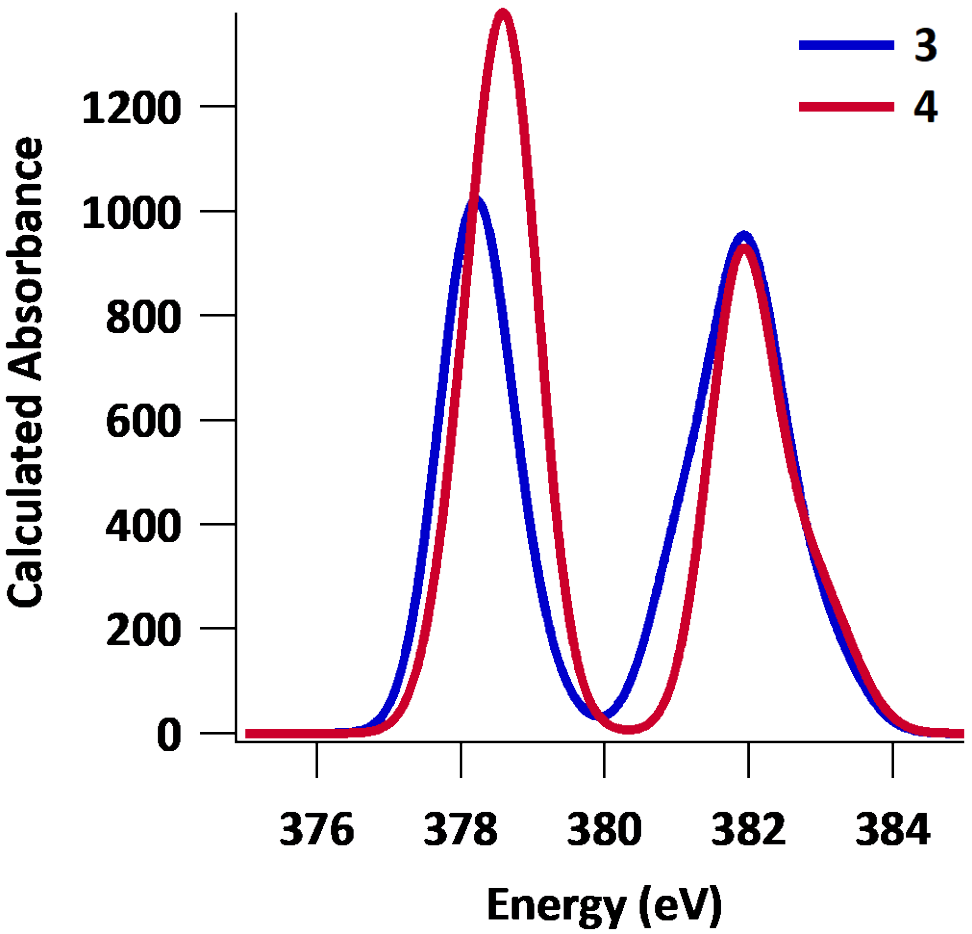

Figure S14. TD-DFT calculated N K-edge XAS for **3** and **4**. Calculations were initiated from spin-unrestricted BP86 single points using the CP(PPP) basis set on Ni and the ZORA-def2-TZVP(-f) basis set on all other atoms. The energy axis is uncorrected. 150 roots were calculated, but insufficient structure is resolved to enable a reliable energy calibration/correction to experimental data.

Figure S15. Correlation of TD-DFT calculated N K pre-edge peak energies to experimental values

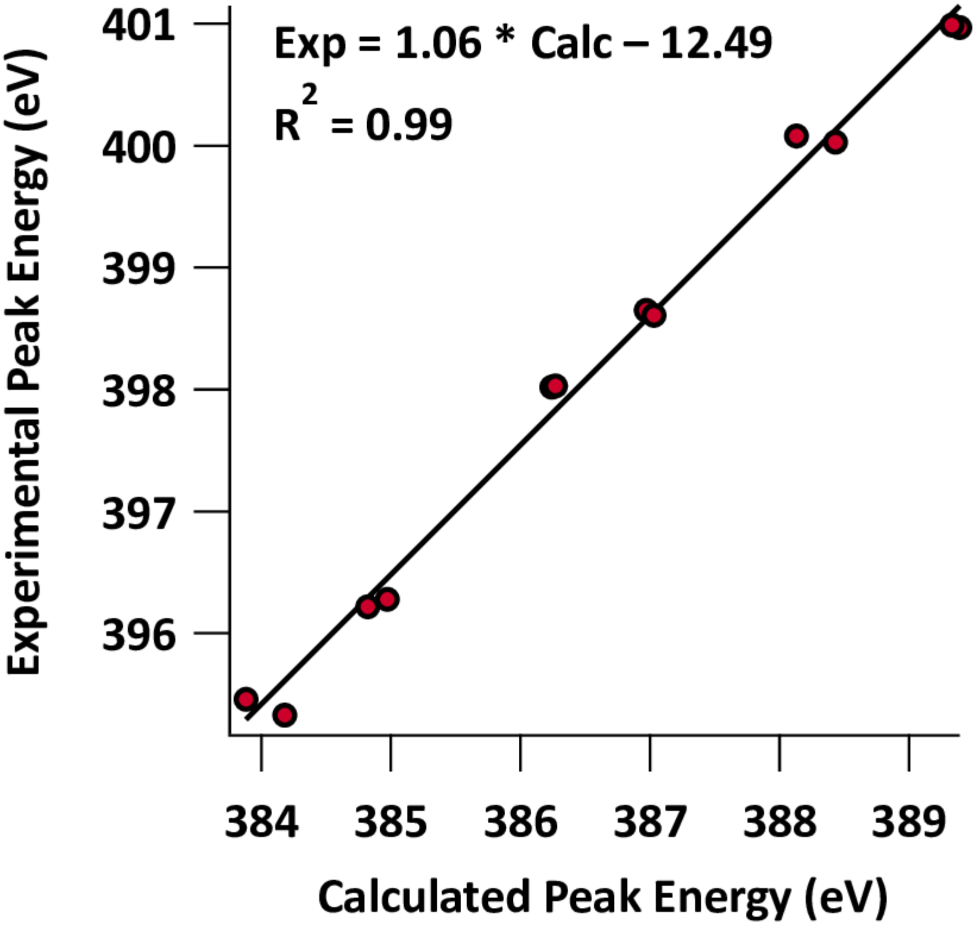

Figure S15. Correlation of TD-DFT calculated N K pre-edge peak energies to experimental values

X-ray Diffraction Techniques

Structures of **1-5** were collected on a Bruker three-circle platform goniometer equipped with an Apex II CCD and an Oxford cryostream cooling device. Radiation was from a graphite fine focus sealed tube Mo Kα (0.71073 Å) source or a I/mS microfocus tube Cu Kα (1.54178 Å) source. Crystals were mounted on a cryoloop or glass fiber pin using Paratone N oil. Structures were collected at 100 K. Data were collected as a series of φ and/or ω scans.

Data were integrated using SAINT[7] and scaled with multi-scan absorption correction using SADABS.[7] The structures were solved by intrinsic phasing, direct methods or Patterson maps using SHELXS-97[8] and refined against F^2 on all data by full matrix least squares with SHELXL-97.[8] All non-hydrogen atoms were refined anisotropically. Hydrogen atoms were placed at idealized positions and refined using a riding model. The isotropic displacement parameters of all hydrogen atoms were constrained to be 1.2 times the parameter of the atoms they were linked to (1.5 times for methyl groups). Further details on particular structures are noted below.

(^{AdF}L)NiCl(py)$_2$ (1): The structure was solved in the monoclinic space group $P2_1/c$ with four molecules per unit cell and one molecule in the asymmetric unit. The asymmetric unit also contained one molecule of disordered hexane, in which the crystal was grown. Due to apparent low occupancy and high disorder, an acceptable model could not be refined. A solvent mask was applied to reduce unrefined electron density. (CCDC 1953729)

(^{AdF}L)Ni(py) (2): The structure was solved in the monoclinic space group $P2_1/c$ with four molecules per unit cell and one molecule in the asymmetric unit. (CCDC 1953730)

(^{AdF}L)Ni(NMes) (3): The structure was solved in the triclinic space group $P\bar{1}$ with two molecules per unit cell and one molecule in the asymmetric unit. The asymmetric unit also contained one molecule of disordered hexane, in which the crystal was grown. Due to apparent low occupancy and high disorder, an acceptable model could not be refined. A solvent mask was applied to reduce unrefined electron density. (CCDC 1953731)

(^{AdF}L)Ni(NAd) (4): The structure was solved in the orthorhombic space group $Pna2_1$ with four molecules per unit cell and one molecule in the asymmetric unit. The molecule displays full molecule disorder. Cracking of the crystal even under cryogenic stream was observed. Due to the

low crystal quality, several atoms were refined using thermal ellipsoid restrains and constraints. (CCDC 1953732)

(^{AdF}L)Ni(NHAd) (5): The structure was solved in the monoclinic space group $P2_1/c$ with four molecules per unit cell and one molecule in the asymmetric unit. The asymmetric unit also contained one molecule of disordered hexane, in which the crystal was grown. Due to apparent low occupancy and high disorder, an acceptable model could not be refined. A solvent mask was applied to reduce unrefined electron density. The hydrogen atom connected to amide nitrogen was located on the difference map. The completeness is 95% due to geometric restriction of using Cu source. (CCDC 1953733)

Table S1. X-ray Diffraction Experimental Details

	$(^{AdF}L)NiCl(py)_2$ (1)	$(^{AdF}L)Ni(py)$ (2)	$(^{AdF}L)Ni(NMes)$ (3)
**Moiety Formula**	$C_{45}H_{44}ClF_5N_4Ni$	$C_{40}H_{39}F_5N_3Ni$	$C_{44}H_{45}F_5N_3Ni$
**FW**	830.00	715.45	769.54
**Crystal System**	Monoclinic	Monoclinic	Triclinic
**Space Group (Z)**	$P2_1/c$	$P2_1/c$	$P\bar{1}$
**a (Å)**	19.2423(8)	17.7017(3)	9.5949(7)
**b (Å)**	10.9261(5)	15.1269(2)	11.8426(10)
**c (Å)**	21.5899(10)	13.1419(2)	18.3253(14)
**α (°)**	90	90	92.953(3)
**β (°)**	106.046(2)	109.572(2)	104.293(2)
**γ (°)**	90	90	100.626(2)
**Volume ($Å^3$)**	4362.3(3)	3315.5(1)	1972.9(3)
**Calc. ρ (mg/m^3)**	1.264	1.433	1.295
**μ (cm^{-1})**	1.674	1.374	0.549
**Crystal size (mm)**	0.26×0.15×0.11	0.42×0.21×0.08	0.26×0.20×0.03
**Reflections**	9954	9959	9938
**Completeness (to 2θ)**	0.994	0.966	0.997
**GOF on F^2**	1.031	1.036	1.044
**R_1, wR_2^c [I > 2σ(I)]**	0.0443, 0.1273	0.0444, 0.1266	0.0517, 0.1477

	$(^{AdF}L)Ni(NAd)$ (4)	$(^{AdF}L)Ni(NHAd)$ (5)
**Moiety Formula**	$C_{45}H_{49}F_5N_3Ni$	$C_{45}H_{50}F_5N_3Ni$
**FW**	785.58	786.59
**Crystal System**	Orthorhombic	Monoclinic
**Space Group (Z)**	$Pna2_1$	$P2_1/c$
**a (Å)**	14.1294(9)	12.0799(4)
**b (Å)**	16.6197(8)	14.4604(5)
**c (Å)**	15.8282(8)	24.1769(10)
**α (°)**	90	90
**β (°)**	90	91.408(2)
**γ (°)**	90	90
**Volume ($Å^3$)**	3716.9(4)	4274.5(3)
**Calc. ρ (mg/m^3)**	1.404	1.222
**μ (mm^{-1})**	1.275	1.109
**Crystal size (mm)**	0.12×0.08×0.02	0.16×0.12×0.03
**Reflections**	9905	4358
**Completeness (to 2θ)**	0.966	0.949
**GOF on F^2**	1.037	1.036
**R_1, wR_2^c [$I > 2\sigma(I)$]**	0.0792, 0.2265	0.0444, 0.1266

Figure S16. Solid-state structure of (^{AdF}L)Ni(py) (1)

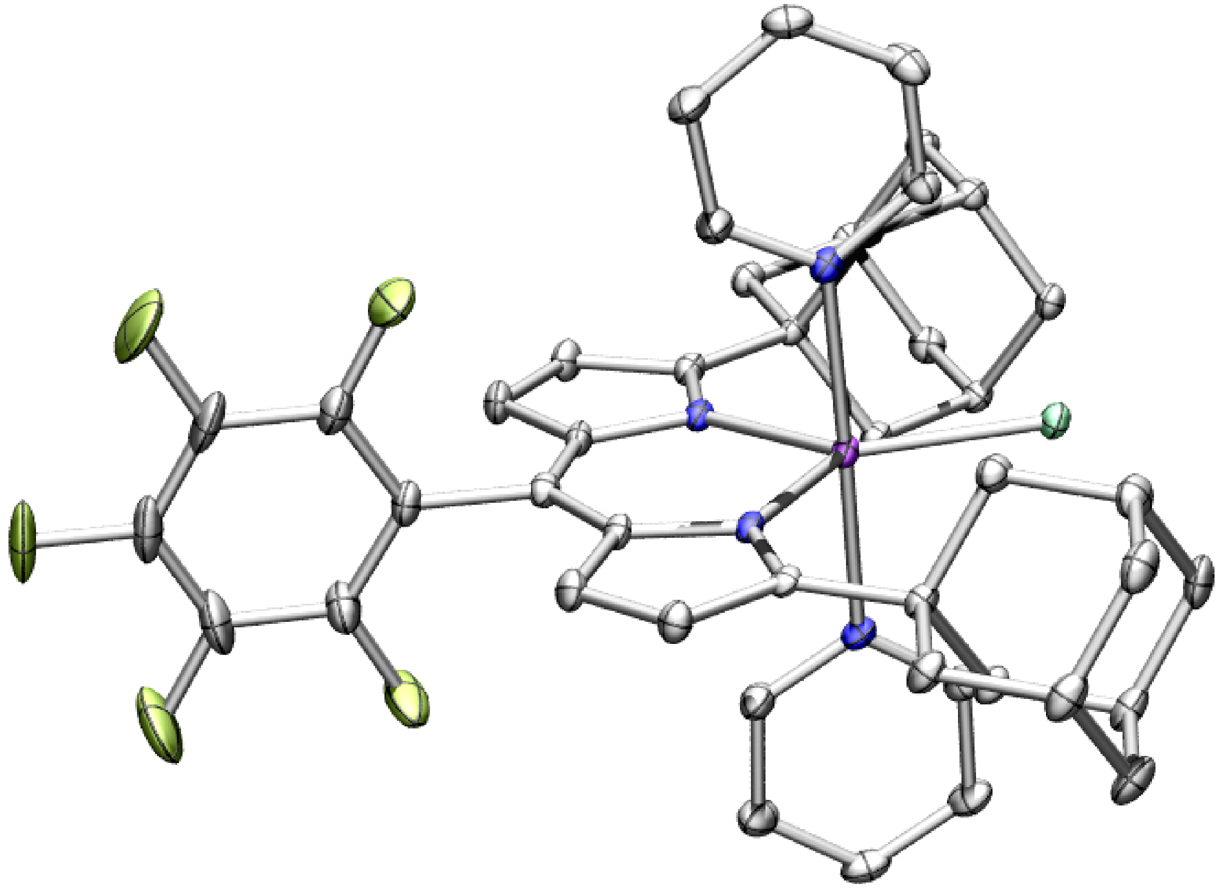

Figure S16. Solid-state molecular structure for (^{AdF}L)NiCl(py)$_2$ (**1**) with thermal ellipsoids at 50% probability level. Hydrogen atoms omitted for clarity. nickel = purple, nitrogen = blue, carbon = gray, chlorine = green, fluorine = yellowgreen.

Figure S17. Solid-state structure of (^{AdF}L)Ni(py) (2)

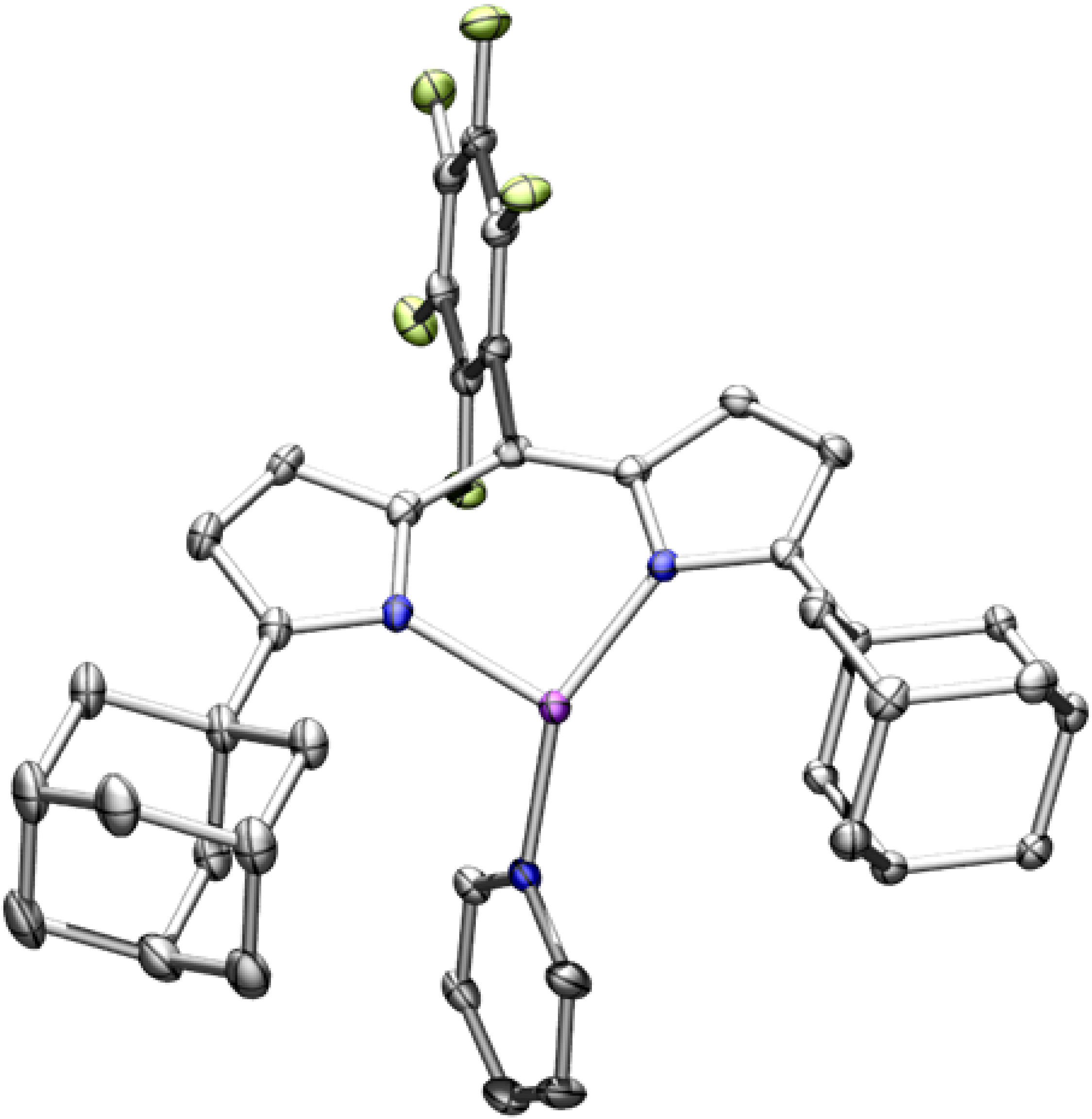

Figure S17. Solid-state molecular structure for (^{AdF}L)Ni(py) (**2**) with thermal ellipsoids at 50% probability level. Hydrogen atoms omitted for clarity. nickel = purple, nitrogen = blue, carbon = gray, fluorine = yellowgreen.

Figure S18. Solid-state structure of (^{AdF}L)Ni(NMes) (3)

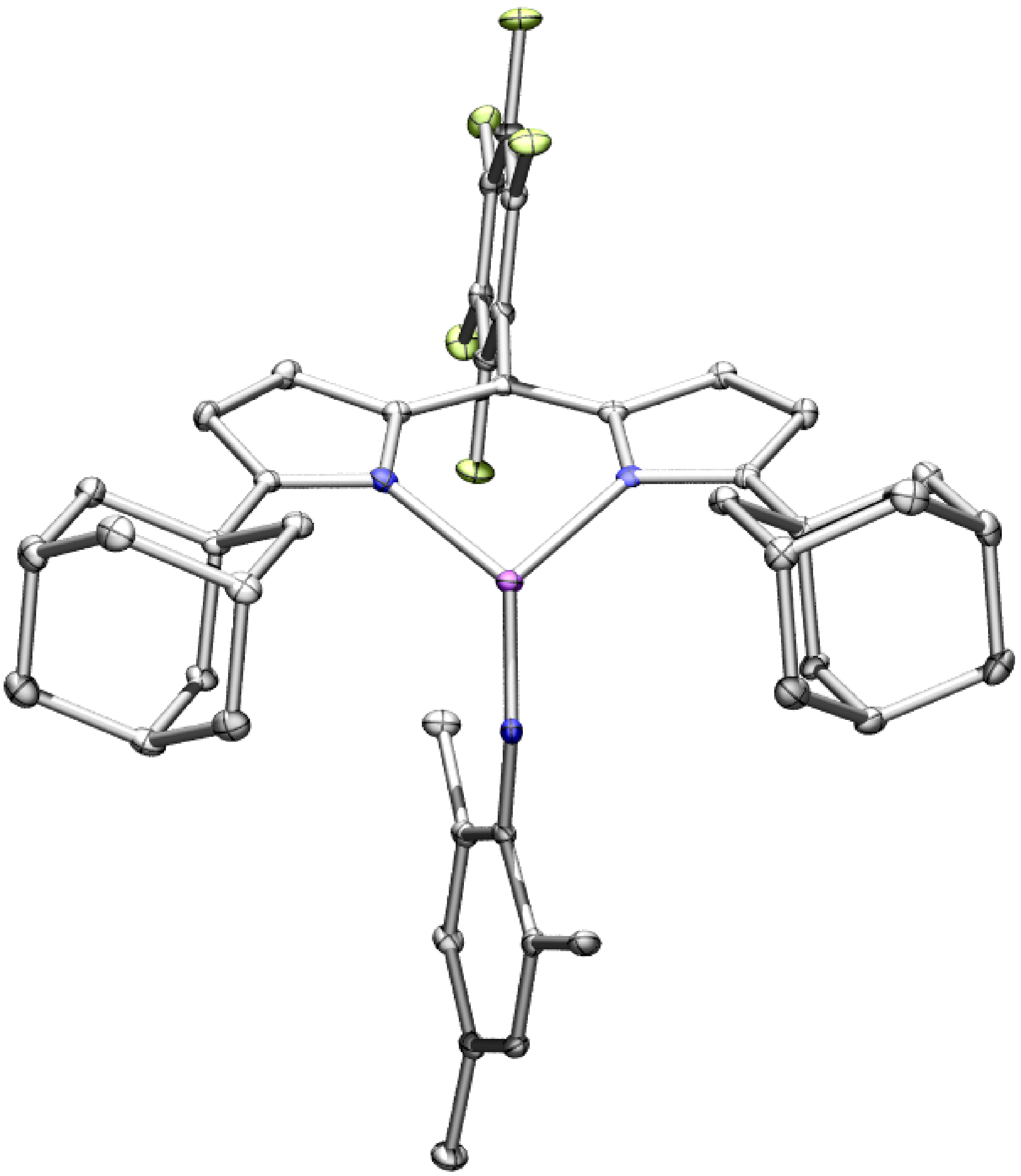

Figure S18. Solid-state molecular structure for (^{AdF}L)Ni(NMes) (**3**) with thermal ellipsoids at 50% probability level. Hydrogen atoms omitted for clarity. nickel = purple, nitrogen = blue, carbon = gray, fluorine = yellowgreen.

Figure S19. Solid-state structure of (^{AdF}L)Ni(NAd) (4)

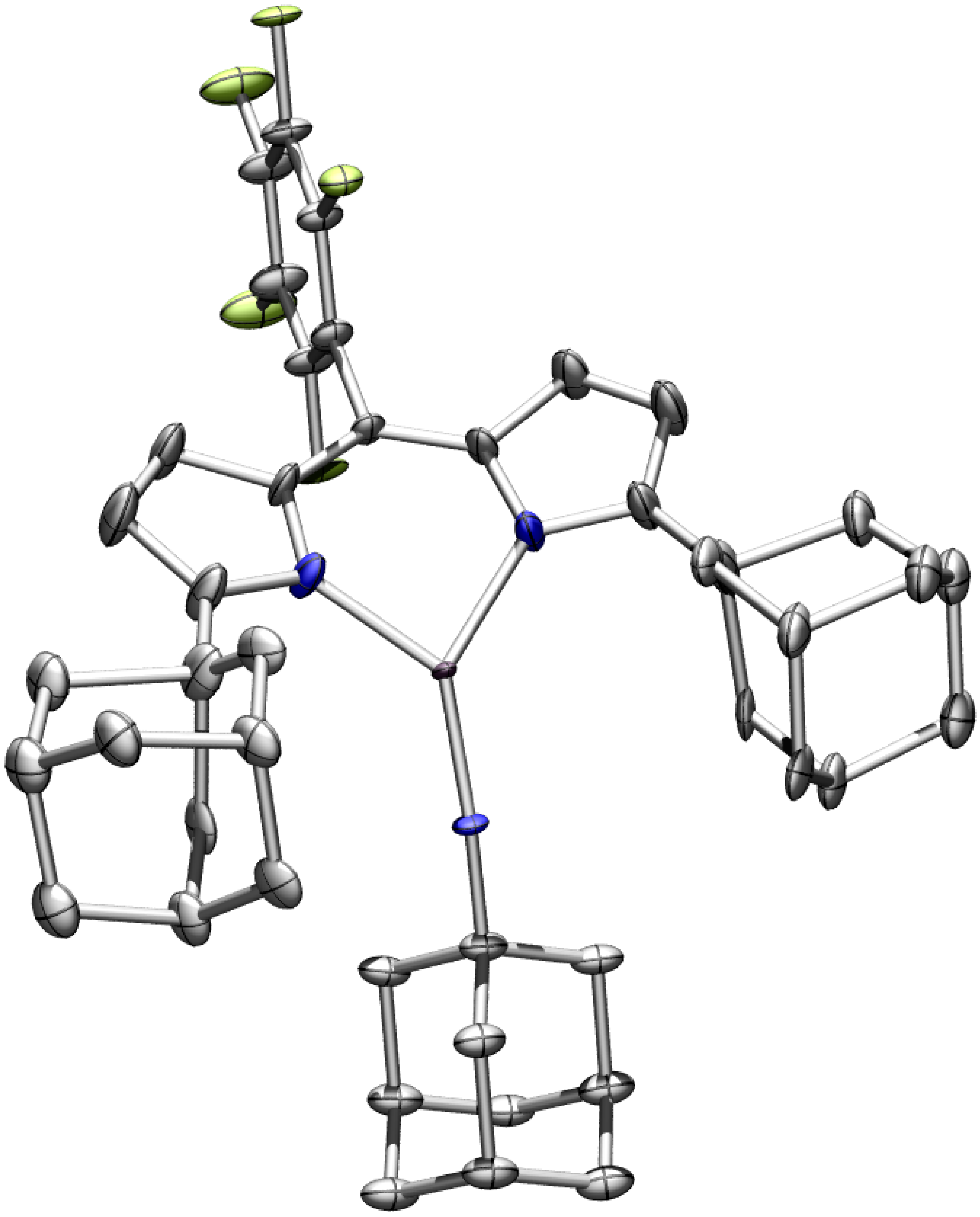

Figure S19. Solid-state molecular structure for (^{AdF}L)Ni(NAd) (**4**) with thermal ellipsoids at 30% probability level. Hydrogen atoms omitted for clarity. nickel = purple, nitrogen = blue, carbon = gray, fluorine = yellowgreen. One of the three molecules from the asymmetric unit shown.

Figure S20. Solid-state structure of (^{AdF}L)Ni(NHAd) (5)

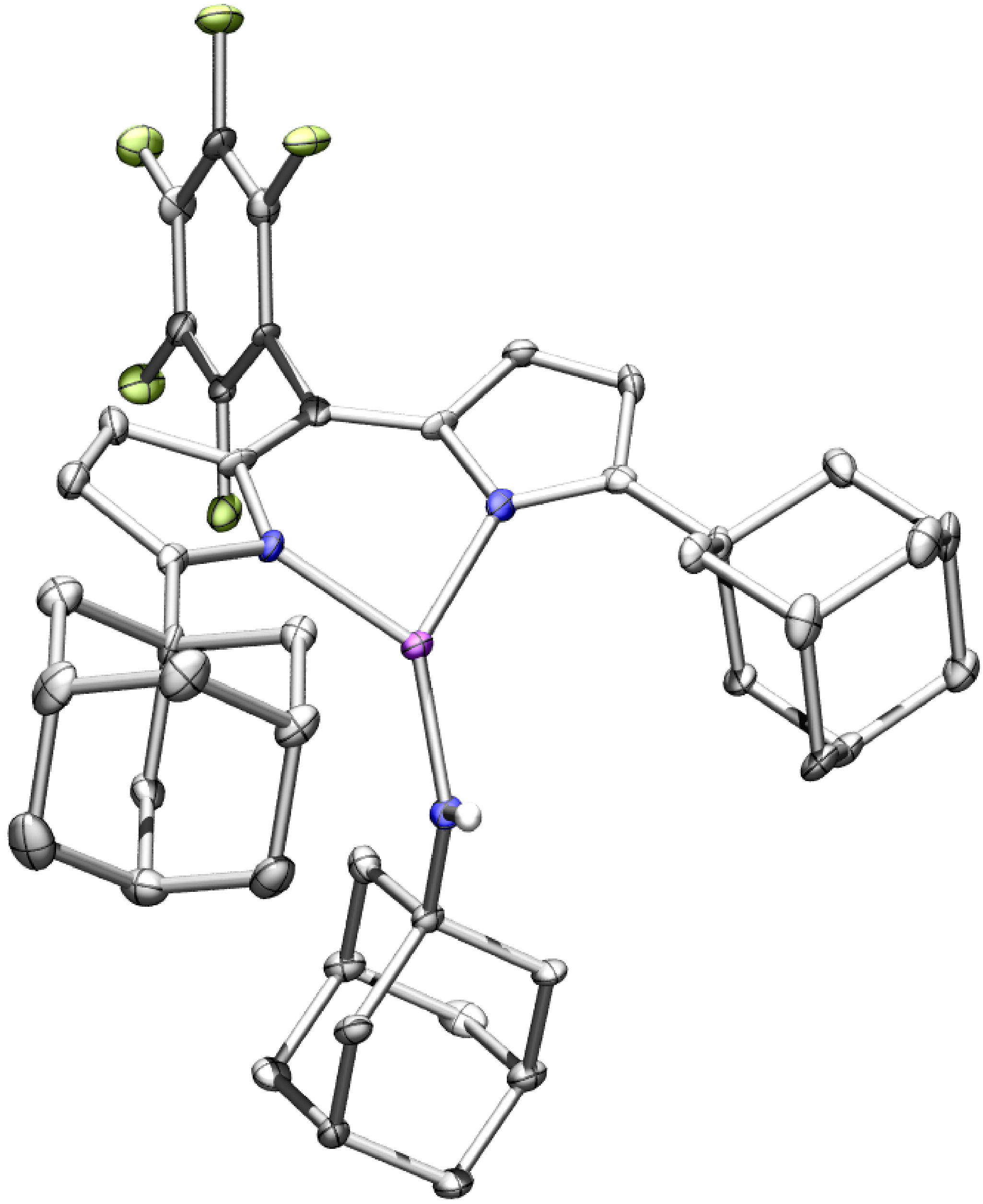

Figure S20. Solid-state molecular structure for (^{AdF}L)Ni(NHAd) (**5**) with thermal ellipsoids at 50% probability level. Hydrogen atoms (except for one connected to amide nitrogen) omitted for clarity. nickel = purple, nitrogen = blue, carbon = gray, fluorine = yellowgreen.

References

1. Wilding, M. J. T.; Iovan, D. A.; Betley, T. A., High-spin iron imido complexes competent for C–H bond amination. *J. Am. Chem. Soc.* **2017**, *139,* 12043-12049.
2. Ekennia, A. C.; Onwudiwe, D. C.; Ume, C.; Ebenso, E. E., Mixed ligand complexes of N-methyl-N-phenyl dithiocarbamate: synthesis, characterisation, antifungal activity, and solvent extraction studies of the ligand. *Bioinorg. Chem. Appl.* **2015**, *2015,* 10.
3. Stoll, S.; Schweiger, A., EasySpin, a comprehensive software package for spectral simulation and analysis in EPR. *J. Magn. Reson.* **2006**, *178,* 42-55.
4. Webb, S. M., SIXPack a Graphical User Interface for XAS Analysis Using IFEFFIT. *Phys. Scr.* **2005**, 1011.
5. Chiba, S.; Zhang, L.; Ang, G. Y.; Hui, B. W.-Q., Generation of iminyl copper species from α-azido carbonyl compounds and their catalytic C−C bond cleavage under an oxygen atmosphere. *Org. Lett.* **2010**, *12,* 2052-2055.
6. Wiese, S.; Badiei, Y. M.; Gephart, R. T.; Mossin, S.; Varonka, M. S.; Melzer, M. M.; Meyer, K.; Cundari, T. R.; Warren, T. H., Catalytic C−H amination with unactivated amines through copper(II) amides. *Angew. Chem. Int. Ed.* **2010**, *49,* 8850-8855.
7. *APEX2 Software Suite*; Bruker AXS: Madison, WI, 2009.
8. Sheldrick, G., A short history of SHELX. *Acta Crystallogr., Sect. A: Found. Crystallogr.* **2008**, *64,* 112-122.
9. Neese, F., The ORCA program system. *WIREs Comput. Mol. Sci.* **2012**, *2,* 73-78.
10. Becke, A. D., Density‐functional thermochemistry. III. The role of exact exchange. *J. Chem. Phys.* **1993**, *98,* 5648-5652.
11. Lee, C.; Yang, W.; Parr, R. G., Development of the Colle-Salvetti correlation-energy formula into a functional of the electron density. *Phys. Rev. B* **1988**, *37,* 785-789.
12. Schäfer, A.; Horn, H.; Ahlrichs, R., Fully optimized contracted Gaussian basis sets for atoms Li to Kr. *J. Chem. Phys.* **1992**, *97,* 2571-2577.
13. Schäfer, A.; Huber, C.; Ahlrichs, R., Fully optimized contracted Gaussian basis sets of triple zeta valence quality for atoms Li to Kr. *J. Chem. Phys.* **1994**, *100,* 5829-5835.
14. Weigend, F.; Ahlrichs, R., Balanced basis sets of split valence, triple zeta valence and quadruple zeta valence quality for H to Rn: Design and assessment of accuracy. *Phys. Chem. Chem. Phys.* **2005**, *7,* 3297-3305.
15. Weigend, F., Accurate Coulomb-fitting basis sets for H to Rn. *Phys. Chem. Chem. Phys.* **2006**, *8,* 1057-1065.
16. Neese, F.; Wennmohs, F.; Hansen, A.; Becker, U., Efficient, approximate and parallel Hartree–Fock and hybrid DFT calculations. A 'chain-of-spheres' algorithm for the Hartree–Fock exchange. *Chem. Phys.* **2009**, *356,* 98-109.
17. Kirchner, B.; Wennmohs, F.; Ye, S.; Neese, F., Theoretical bioinorganic chemistry: the electronic structure makes a difference. *Curr. Opin. Chem. Biol.* **2007**, *11,* 134-141.
18. Soda, T.; Kitagawa, Y.; Onishi, T.; Takano, Y.; Shigeta, Y.; Nagao, H.; Yoshioka, Y.; Yamaguchi, K., Ab initio computations of effective exchange integrals for H–H, H–He–H and Mn_2O_2 complex: comparison of broken-symmetry approaches. *Chem. Phys. Lett.* **2000**, *319,* 223-230.
